# The precarious nature of musical encounters in healthcare: An ethnographic multi-case study focusing on the perspectives of care professionals and musicians

**DOI:** 10.1371/journal.pone.0359306

**Published:** 2026-10-05

**Authors:** Monique Bussmann, Marieke Groot, Chris Kuiper, Mieke Cardol

**Affiliations:** 1 Research Centre Innovations in Care, Rotterdam University of Applied Sciences, Rotterdam, The Netherlands; 2 Faculty of Social and Behavioral Sciences, University of Amsterdam, Amsterdam, The Netherlands; Mae Fah Luang University School of Anti Aging and Regenerative Medicine, THAILAND

## Abstract

**Background:**

This paper presents an ethnographic multi-case study exploring how musical encounters in healthcare contribute to experiences of connection, focusing on the perspectives of care professionals and musicians. In addition to studies on music in healthcare that primarily examine music as an auditory stimulus and its effects on care recipients, this study focuses on performative music and considers its impact on the professional level and within the broader organization as well.

**Methods:**

A variety of musical encounters were observed in three healthcare settings. The encounters were examined ethnographically by open observations and open interviews, enhanced by an elicitation technique.

**Results:**

Inductive analysis revealed two major themes: *threshold experiences* and *connective experiences*, organized across three levels: within the musical encounters, among involved professionals, and within the broader organization. These themes comprise a total of 19 subthemes. *Precariousness* emerged as an overarching construct, articulating the dynamics between threshold and connective experiences. These findings demonstrate that musical encounters have the potential to foster connective experiences. However, the occurrence of positive impact appears to be precarious, and this precariousness is twofold: In a positive sense, it reflects the delicacy of the process within the musical encounters, due to their artistic nature. In a negative sense, it highlights the vulnerability of musical encounters, arising from insufficient sustainable support at the organizational level.

**Conclusions:**

To strengthen the potential of musical encounters in healthcare to impact connective experiences, it is recommended to pay greater attention to their precarious nature – by cherishing the delicacy of the encounters themselves, while actively addressing the threats posed on the organizational level.

## 1. Introduction

### 1.1 Context and rationale

In recent years, music has gained increasing relevance in healthcare, both as a tool for improving care and as a subject of research. Musicians are more frequently involved in healthcare settings, and innovative musical practices are being explored in transdisciplinary collaborations [1–4]. In the Netherlands, notable examples include Rotterdam Erasmus Medical Center’s *Music as Medicine* program, the project *Meaningful Music in Healthcare* (*MiMiC)* by Prins Claus Conservatoire in collaboration with Academic Medical Center Groningen, Amsterdam Academic Medical Center’s *Arts in Health* initiative, and the project *Creating Cultures of Care* of HKU University of the Arts*.* Although some programs, such as *MiMiC*, also examine the effects of music on healthcare professionals, most research focuses on its direct impact on patients or other health related target groups, for example through compassionate care [5,6].

The sustainability of healthcare systems is increasingly at risk, requiring structural adaptation. The WHO identifies rising healthcare demands, workforce shortages, and financial pressures as universal challenges [7]. These global trends are evident in the Dutch context, where the Dutch Scientific Council for Government Policy (WRR) warns that rising care demands will outpace economic and demographic growth, threatening the sustainability of the healthcare system [8]. In response, the Dutch government has launched programs to promote labor-saving care models, improve employment practices, and support innovation [9,10]. However, progress remains slow. The Council for Public Health and Society (RVS) advocates shifting focus from technical to social innovation and therefore proposes new forms of collaboration and organization [11]. This perspective is supported by the Ministry of Education, Culture and Science, which stresses the societal value of culture and the need to strengthen the role of creative professionals through cross-sector partnerships [12].

International findings, such as WHO’s scoping review on arts in health [13], demonstrate a substantial evidence base for the health benefits of the arts, but limited policy impact. Therefore, Fancourt and Finn emphasize the need for broader, cross-sector implementation and structural adaptation, including preparing students for collaborative, transdisciplinary work. According to Lewis et al. [14], the potential of the arts to contribute to the quality of care indirectly—for example, by improving healthcare working conditions, enhancing organizational processes, and contributing to the sustainability of the healthcare system—remains underexplored.

Fancourt and Finn [13] identify important indirect forms of impact of the arts on quality of care, such as strengthening caregivers’ clinical skills, mental health and well-being, and understanding of health. Proposing a national agenda *Arts in Health in the Netherlands,* Lewis et al. [14] argue that the arts can strengthen a culture of care by reconnecting professionals with the relational aspects of their work, building resilience, and improving communication with patients. Additionally, Smilde et al. [15] explore how participatory live music can support learning pathways for nurses, moving from professional distance to emotional inclusion and interprofessional reflection. Finally, Van den Berg et al. [5,6] demonstrate that musical encounters enhance compassionate care, by strengthening nurses’ compassion, empathy, and well-being, positively influencing both care delivery and workplace atmosphere [5], as well as nurses’ mood, work pleasure, and team dynamics [6].

### 1.2 View on human beings: Inherently musical and caring

This study is grounded in the view that all human beings are inherently musical, a perspective compellingly argued by Spitzer in *The Musical Human* [16]. Musicality, according to Spitzer, is embedded in deep cognitive structures and manifests through various innate abilities—such as rhythm, pitch recognition, melodic memory, and polyphonic listening. These capacities suggest that everyone has a natural predisposition to engage with and be affected by music. However, the way this musicality is expressed varies across cultures and individuals. While infants are highly receptive to diverse musical expressions, preferences and skills tend to solidify early in life.

Spitzer identifies several qualities that make music particularly valuable in healthcare. Its semantic openness and emotional immediacy allow music to evoke deep feelings. Through *mimesis*, the biological tendency to imitate, music reflects the world and fosters imaginative thinking, enabling individuals to envision possibilities beyond their current reality. Besides, even just listening to music is inherently relational and participatory: the listener mentally imitates the performer, who imitates the composer, who in turn imitates the world, by transforming it into music. Moreover, the human body’s upright posture facilitates shared musical expressions like clapping, singing, and dancing, fostering emotional and social harmony [16].

Concurrently, this study adopts the care ethics framework of Tronto [17], which suggests that all people—including, for example, musicians—are inherently caring. Tronto challenges the notion that care is reserved for professionals or those deemed naturally nurturing. Instead, she emphasizes that everyone is both a caregiver and care receiver throughout life, and that dependency is a fundamental, not deficient, aspect of being human. Care is thus a reciprocal and relational practice [17]. Tronto outlines five phases of care: *caring about* (awareness), *caring for* (responsibility), *caregiving* (action), *care receiving* (responsiveness), and *caring with* (political awareness). These phases are also applicable to musicians’ roles in healthcare, highlighting their potential contributions to care processes.

### 1.3 Ethical and aesthetic perspectives on healthcare

Ethically, this study conceptualizes the allocation of care responsibilities not as a set of fixed roles but as evolving moral practices [17,18]. While Walker [18] politicizes ethics more broadly, Tronto [17] specifically politicizes care ethics, particularly in relation to how care responsibilities are allocated within society. From this perspective, musicians’ involvement in healthcare can be seen as introducing new voices into these negotiations, potentially reshaping the distribution of care responsibilities and contributing to a more sustainable system.

Aesthetically, this study values musical encounters not only for their sensory impact. The care process itself can be regarded as having aesthetic properties as well [19,20], as can organizational processes [21]. Furthermore, this study values musical encounters for their transformative potential. Recognizing this potential can enhance the quality of care and job satisfaction in healthcare, and reveal deeper insights—such as questions of justice—within care settings and society [19,20]. Drawing on Schiuma [21], this study assumes that leveraging the aesthetic dimension of organizations can foster institutional adaptability. Artistic practices, including musical ones, offer emotional knowledge that complements technical expertise, reinforcing the human-centered nature of, for example, healthcare institutions and legitimizing partnerships with artists such as musicians [21].

### 1.4 Research question

This study investigates the following question: *In what way do musical encounters in healthcare enhance experiences of connection focusing on the perspectives of care professionals and musicians?*

#### Understandings.

A musical encounter refers to an artistic interaction between musicians and individuals in healthcare, centered on making music. The focus lies beyond music therapy or passive listening (e.g., background music), emphasizing the relational and experiential dimension of live music. Live music in healthcare settings offers unique benefits that go beyond recorded music. As Van der Wal-Huisman [22], drawing on Brown and Knox [23] and Swarbrick et al. [24], highlights, live music fosters social interaction, shared experiences, and engagement, while also offering a visual and emotional presence. Its adaptability—whether in sound, intensity, or content—allows musicians to tailor performances to the specific needs of patients and environments. Importantly, live music does not require prior musical skills from participants and can be experienced passively or actively, depending on the individual’s condition and preferences [25,26].

Fancourt and Finn’s [13] logic model linking the arts with health, identifies several health-promoting components. While social interaction is an obvious form of connection, the model also points to broader forms of connectivity. Artistic experiences can link individuals not only to others, but also to their emotions, imagination, sensory awareness, cognition, physicality, and even their healthcare environment and treatment. These dimensions, supported by studies such as Juslin [27] and Fancourt et al. [28], suggest that musical encounters can foster deep and multifaceted experiences of connection.

## 2. Materials and methods

Taking an interpretive stance, this study employs an ethnographic multiple-case study design, a qualitative approach drawing on multiple sources of evidence to ensure a rich, contextualized understanding of real-life phenomena. The ethnographic lens emphasizes understanding how community members interpret their experiences, requiring the researcher to build relationships while maintaining enough distance to avoid influencing the natural dynamics [29].

### 2.1 Case selection and participant recruitment

Three cases were selected through convenience sampling, leveraging the researcher’s network of musicians in healthcare settings (Table 1). The first case involved Bedside Music, a collective of cellists dedicated to person-centered performances in healthcare environments. The researcher, already familiar with its artistic leader, was introduced to a general hospital that had recently partnered with the collective. The second case emerged from a collaboration, initiated by the researcher, between a music therapist at an academic medical center and a postdoctoral researcher at a local conservatory. The third case featured a joint music initiative between a residential care center and a local arts and culture institution, which was looking for a researcher via an online platform for social arts projects. Although not exhaustive, the varied nature of these cases offers a broad perspective on musical encounters in healthcare and social domain. The individual participants (such as project leaders, care professionals and musicians) were recruited by the respective facilitators, provided with information about the study, and invited to participate voluntarily.

**Table 1:** Overview cases: Description of musical encounters.

Where?	What?	Who?	When?
**Case 1** General hospital and Beside Music	Person centered music made by two cellists playing on one cello, while moving through the hospital to perform in wards and corridors.	Cellists of Bedside Music; Patients, visitors and staff.	Every other week, based on a sequence of yearly arrangements.
**Case 2** Academic medical center and a conservatory	**Musical encounter 1** Several musical performances in the entrance hall on Nursing Day.	Students and teachers of the conservatory; Patients, visitors and staff waiting or passing by.	One-time.
	**Musical encounter 2** A singing session as a team building activity for care professionals, facilitated by a vocal leader (conservatory alumnus).	Vocal leader; Members and team leader of the hospital’s psychosocial care team.	One-time.
	**Musical encounter 3** Music students coaching hospital employees preparing for an internal concert by and for colleagues.	Students and teachers of the conservatory; Hospital staff who perform or attend the concert.	Yearly, over a period of a few months from kick-off to concert.
**Case 3** Residential care center and a center for arts and culture	Choir singing at two different care locations, led by two professional conductors, working towards biannual concerts.	Choir conductors delegated by the center for arts and culture; Residents, relatives, welfare and care staff of the care center; Managers and project leaders of both institutions.	A two-year granted project.
			Location 1: weekly, for two years.
			Location 2: weekly for one year (out-roll).

### 2.2 Data collection

Data were collected over a two year period, solely by the researcher and mainly through open observations and interviews, supplemented by an elicitation technique (Table 2). Key participants included musicians, care staff and project leaders/facilitators across the three cases. Observations were conducted during musical events such as performances, choir sessions, and meetings. The researcher participated modestly in activities (e.g., by singing along or assisting participants in turning pages) or observed unobtrusively to preserve the authenticity of the encounters. Notes were taken when possible, or voice recordings were made post-event. In cases where COVID-19 restrictions prevented observation, participating musicians, welfare and care professionals submitted reflection reports. Open interviews were conducted with key figures from each project. To deepen emotional insight, the researcher used an “Emotional process form”, adapted from *Nonviolent Communication* principles [30]. This form, which was pilot tested during a project team meeting, prompted participants to list five sequential emotional keywords reflecting their recent experiences, which then served as a basis for the conversation. The form encouraged self-empathy and reflection, enhancing the depth of the interviews.

**Table 2:** Database: Cases and Data.

Cases	Data collection
General hospital and Bedside Music	**Performances Bedside Music** Observation reports performances¹ (n = 3) Recorded reflection reports cellists (n = 6) Recorded interviews cellists² (n = 6) Recorded interviews project leader² (n = 2) Recorded group interview team leaders² (n = 7)
Academic medical center and a conservatory	**Performances on Nursing Day** Observation report performances¹ (n = 1) Reflection report musician (n = 1) Observation report evaluation meeting¹ ² (n = 3)
	**Singing session care team** Observation report singing session¹ (n = 1) Emotional process form singers (n = 28) Recorded interviews singers (n = 4) and vocal leader² (n = 2)
	**Concert by and for colleagues** Observation reports kick-off and concert¹ (n = 2) Observation report concert announcements on the hospital’s intranet (n = 1) Observation report e-mail updates project leaders¹ (n = 1) Recorded interviews project leaders¹ ²(n = 3) Evaluation forms musicians (n = 17) and audience (n = 68)
Center for residential elderly care and a Center for arts and culture	**Choir singing** Observation reports rehearsals and concerts¹ (n = 7) Observation reports care team, project and management team meetings¹ (n = 16) Reflection reports welfare professionals (n = 10) and conductor (n = 7) Recorded interviews project leaders and involved musicians and care professionals² (n = 27)

^1^= by the researcher; ^2^ = emotional process form included;

n = number of documents (recordings, reports, forms etc.)

Interviews lasted 30–60 minutes and were mostly conducted online due to pandemic constraints. Within each case, data collection continued until no substantive new themes emerged in several of the observations and interviews, or until institutional access restrictions due to the COVID-19-pandemic made further fieldwork impossible.

### 2.3 Data analysis

The data were compiled into a single database and qualitatively analyzed using Atlas.ti. The analysis followed an inductive process of constant comparison [29]: open coding identified meaningful occurrences and participant interpretations, followed by axial coding, using the group code function in Atlas.ti. This process yielded 19 subthemes, which were organized into a theme matrix, comprising two major themes (columns)—*threshold experiences* and *connective experiences*—across three levels (rows): within the musical encounters, among involved professionals, and within the broader organization. The researcher conducted the coding and discussed interpretations with two supervisors. The process was thoroughly documented to ensure transparency and traceability. All data—including interviews, observations, reports, and forms—as well as all analysis steps were securely stored in SURF Research Drive. Metadata were published on the repository platform DataverseNL (https://dataverse.nl/dataset.xhtml?persistentId=doi:10.34894/FBHF2Z). In accordance with the platform’s decision tree (https://zenodo.org/records/18390982), the data were assessed and deemed ineligible for publication, therefore only the metadata were published. The COREQ checklist [31] was used to assess reporting quality and the generative AI tool Microsoft 365 Copilot (bizchat.20251016.51.2) was used to check the manuscript for English grammar.

### 2.4 Fieldwork

The study coincided with the COVID-19 pandemic, which demanded the full attention of healthcare institutions and made them highly reluctant to grant access to musicians and, even more, to researchers. Consequently, the researcher’s access for data collection was largely limited to musicians, project leaders/facilitators and directly involved staff members, rather than to patients/residents, care staff and managers.

As a result, the dataset does not include the direct voices of patients/residents and healthcare professionals who were not directly involved, or board members. However, the musicians, project leaders/facilitators, and care professionals who participated directly proved both willing and able to represent the perspectives of patients/residents, colleagues, and managers. It can be concluded that this has limited the ability to answer the research question with the desired comprehensiveness. Nevertheless, these specific circumstances provided particularly rich insights into the dynamics at the professional level, as well as at the other levels.

Being a former nurse and project manager in healthcare, as well as an amateur musician herself, the researcher is acquainted with the worlds of experience of respondents and capable of getting access, in-depth observation and interviewing, analyzing, and interpreting. As an experienced qualitative researcher, she continuously reflected on which attitude would best preserve the naturalness of the situation, and remained aware that, despite these efforts, her presence may have influenced the participants’ behaviour.

### 2.5 Ethical considerations

The research was conducted in accordance with the European General Data Protection Regulation (GDPR) and the Netherlands Code of Conduct for Research Integrity of the Royal Netherlands Academy of Arts and Sciences [32]. Rotterdam University of Applied Sciences (RUAS) and its researchers are committed to this code of integrity, having signed it.

Although RUAS aims for research data to be publicly available wherever possible, according to the prevailing data protection and confidentiality requirements, researchers of the university are not permitted to publicly share full transcripts or field notes, because of its contextual richness which makes full de-identification impossible without severely compromising the scientific value of the dataset and the participants’ consent.

The musical encounters were initiated as innovative activities by the participating care institutions, rather than as a study intervention. Consequently, the researcher did not intervene in the natural setting, which is consistent with the ethnographic research design. As no data were collected directly from patients or residents, and no personal or health-related data were gathered at all, the study did not require medical-ethical approval. At the time the research design was reviewed (2019), RUAS did not yet have a Research Ethics Committee. The secretary of the current committee can be approached with requests for data access (e-mail: eco@hr.nl).

The project leaders/facilitators of the participating institutions informed the participants—both orally and in writing—about the researcher’s involvement in the music project. They also introduced the researcher to the participants, giving them the opportunity to receive more context-specific information and to refrain from participating. At the beginning of all interviews, the researcher checked if the participant recollected the information. Next, she asked the respondent to provide verbal consent to the use the interview recording research purposes, ensuring the participant all information would be anonymized and safely stored. All verbally informed consents were captured in the recordings of the interviews, that in most cases were conducted online due to COVID-19-measures. Finally, for ethical and validating purposes, the facilitators in each case were given the opportunity to provide feedback on the findings and on a draft version of the manuscript. After their minimal feedback was processed, they all provided permission for publication.

## 3. Results

As described in the Methods and Materials section, the analysis process resulted in a theme matrix consisting of 19 subthemes. These subthemes have been organized into two themes, *threshold experiences* and *experiences of connection,* and distributed across three levels of occurrence: *within the musical encounters*, *among involved professionals* and *within the broader organization*. Reflecting on this matrix, *precariousness* emerged as the overarching construct.

### 3.1 Experiences within musical encounters

Musical encounters in healthcare settings appear to lead to various connective experiences among individuals involved. However, these experiences are often preceded by threshold experiences that must be navigated before connective experiences can occur (Table 3).

**Table 3:** Threshold and connective experiences within musical encounters.

Experiences of connection within musical encounters	Threshold experiences *(uncomfortable feelings)*	Connective experiences *(personal sensations)*
	Listeners: • sensation of unexpectedness or elusiveness • diversity in musical preferences and abilities Musicians: • healthcare as ‘unfamiliar destination’ • deployment of sensitivity and flexibility • commitment to professionalism and artistry	• connection with others, with life or, with the body • connective atmosphere or musical enjoyment

#### 3.1.1 Threshold experiences of listeners and musicians.

*Unexpectedness and elusiveness (among listeners):* Especially in hospitals musical performances can feel unexpected, sometimes leading to uncomfortable feelings among listeners (patients/residents, visitors, and professionals), such as embarrassment. Care professionals may find musical performances disruptive, particularly if they interfere with their workflow. Some feel uncertain about whether the professional setting allows for active engagement.

*“Singing requires a different kind of safety. I asked myself: ‘Do I feel okay enough here to actually do this now?”’ (healthcare professional)*

Musical encounters are often described as magical or elusive: They may be fleeting or intense, and can take unexpected turns, such as patients asking the musicians what the performance means to them personally. This unpredictability may feel uncomfortable. Musicians, listeners, and facilitators recount surprising moments of connection after surrendering to these feelings, describing a sense of ‘being taken along’.

*Musical preferences and abilities (among listeners):* Especially in multi-patient rooms, musicians must navigate differing musical tastes. While some listeners enjoy the warm tones of a cello, others find it too melancholic. Some care professionals seek calm music to relax; others prefer upbeat music that matches their work rhythm.

*“I hear two nurses saying the music isn’t to their taste… One says she’d prefer something more cheerful to lighten the atmosphere.” (observation researcher)*

*Unfamiliar destination (musicians):* Musicians often experience healthcare institutions as unfamiliar and intimidating. They may feel invisible, performing for listeners who seem absorbed in their own bubble.

*“I really enjoyed it; however, when I entered and my music student was performing, it felt somewhat as though she was playing in the main hall of a central railway station. It was not apparent that anyone was actually listening.” (conservatory teacher)*

Cellists from Bedside Music mention that they sometimes experience anxiety before crossing the physical threshold of patient rooms, out of concern to be disruptive.

*Sensitivity and flexibility (musicians):* Musicians must quickly assess the mood and preferences of listeners, adjusting their repertoire accordingly. Choir conductors navigate the boundaries of how far they can go in stimulating elderly singers, balancing encouragement with acceptance. One conductor describes how unexpected reactions—such as a singer offering him a kiss—initially overwhelmed him but later became opportunities for growth.

*Professionalism and artistry (musicians):* Musicians maintain high standards for acoustics, concentration, artistic autonomy and performance quality. Yet, these ideals may conflict with situational constraints in healthcare environments. One choir conductor felt that feedback from care professionals compromised his sense of craftsmanship, while another emphasized the importance of a learning curve in the singers as a reflection of his professional expertise.

#### 3.1.2 Listeners’ and musicians’ experiences of connection.

*Connection with others, with life, or the body:* Cellists from Bedside Music observe that shared musical experiences—listening, singing, clapping, and expressing emotions—create bonds among patients. These connections are visible in the synchronicity of reactions and shared attention. Care professionals report seeing patients more as individuals thanks to the musical performances, and vice versa. Staff concerts strengthen collegial ties across departments and positions and connect employees closer to the institution.

Musicians experience that their performances reconnect individuals with their personal histories and emotions. Patients often feel linked to life beyond illness, while care professionals may reconnect with their private lives and feelings. Welfare professionals at the residential care center, report that choir singing evokes childhood memories and stimulates reflections on life and death.

*“The older you get, the more you reach back to your past, including your musical past.” (welfare professional)*

As observed by the researcher, music activates listeners physically, even those in poor health. Spontaneous movements—such as toe-tapping, singing, or conducting—are common. Elderly choir singers are energized by rehearsals, while care professionals feel uplifted by the singers’ enthusiasm. Music also offers a non-verbal outlet for expression, described by one care professional as ‘bringing air into the lungs’.

*Connective atmosphere and musical enjoyment:* Team leaders note that Bedside Music helps break through tension or gloom on the ward, creating a cozy, comforting, and relaxed atmosphere. Welfare professionals emphasize that the positive atmosphere encourages the free expression of elderly singers. Listeners enjoy both the musical performances and the musicians’ joy in performing. Care professionals especially appreciate seeing patients respond positively, though they may feel uneasy when music is directed at them. Staff concerts evoke emotions such as pride, admiration, and togetherness.

*“It was such a positive experience… I really enjoyed the music.” (healthcare professional)*

#### 3.1.3 Interpretations and implications.

The threshold experiences within the musical encounters could be summarized as *uncomfortable feelings*, and the connective experiences as *personal sensations of connection*. Although uncomfortable feelings often precede connective sensations, they are not a necessary precondition. Connections frequently occur without a preceding threshold experience. At the same time, initial uncomfortable feelings can act as a barrier to connection; conversely, they may also enhance and deepen them. Within musical encounters, most listeners appear willing and motivated to overcome their initial uneasiness and to engage in connective experiences.

An empirical limitation is the absence of patients’ and residents’ direct voices, since the findings are based on the musicians’ and care professionals’ experiences and observations regarding musical performances, supplemented by the researcher’s observations. Nevertheless, this limitation is somewhat offset by the wealth of existing studies that have already documented patients’ and residents’ perspectives on the role and effects of music in care settings [1–4].

### 3.2 Experiences among involved professionals

Musical encounters foster professional connections as well. Also these connections are often preceded by threshold experiences (Table 4).

**Table 4:** Threshold and connective experiences among involved professionals (in care and music).

Experiences of connection among involved professionals	Threshold experiences *(professional differences)*	Connective experiences *(professional enrichment)*
	• differences in personality, experience, approach, or role perception • differences in musical preferences or abilities • concerns, embarrassment, or work pressure	• connection between professionals and patients/residents, between disciplines, or between musicians and care professionals • connective musical involvement, job satisfaction, inspiration and enthusiasm

#### 3.2.1 Professionals’ threshold experiences.

*Differences in personality, experience, approach, or role perception:* At the residential care center, collaboration between two choir conductors become strained. The more experienced and extraverted conductor feels disappointed that his colleague does not utilize his expertise, while the less experienced and more introverted conductor prefers to develop his own style independently. Care managers and welfare professionals have their own opinions about the conductors’ approaches, who accentuate either a joyful atmosphere or musical development.

Welfare professionals notice that choir rehearsals continue to be seen as a welfare activity by care professionals, which hinders the intended integration of care and welfare.

*“We saw staff helping out—bringing people in, staying around—but they didn’t report anything, not even after the concert.” (welfare professional)*

*Differences in musical preferences or abilities:* Some hospital team leaders are initially skeptical about Bedside Music because they have little affinity with classical music. However, they later observe that the musical performances have a calming effect, and that the predominantly older patients appreciate them. At the residential care center, care professionals believe music should be brought closer to residents who are unable to attend rehearsals.

*Concerns, embarrassment or work pressure:* Care managers at the residential care center initially express concern that residents’ family members would be skeptical about their relatives participating in a choir and performing in concerts. They also worry about the additional effort required from care professionals to support choir rehearsals and concerts. However, the infectious enthusiasm and professionalism of the conductors help them alleviate their concerns. Care professionals often feel reluctant integrating music into their care practices, as they do not consider themselves musical or feel embarrassed to sing. They appreciate that choir rehearsals take choir members away from the unit, offering care staff a moment of relief. They emphasize that integrating music into care practices should not increase their workload but rather reduce it.

#### 3.2.2 Professionals’ experiences of connection.

*Connection between professionals and patients/residents, across care disciplines, or between musicians and care professionals:* A participant in a care team singing session highlights the importance of stepping outside one’s comfort zone as a care professional: experiencing vulnerability and uncertainty—similar to what patients feel—can deepen empathy and support person-centered care.

Welfare professionals at the residential care center aim to collaborate with care staff by sharing observations and helping integrate music into daily care routines. The center’s music therapist sees opportunities from his professional position to enhance the well-being of care professionals and to strengthen mutual interprofessional support within the care process. Musicians from Bedside Music actively seek to involve care professionals in performances to increase their engagement with, and support for, the performances. The conductors of the senior choirs value collaboration with welfare professionals, who know the singers better, facilitate rehearsals, and provide feedback. Welfare professionals learn from a conductor’s respectful approach while elevating the elderly to a higher musical level.

*“We’re not able to get the elderly singing a round during an ordinary singing afternoon, but the conductor manages to do it. We can learn from that too. Maybe we could try it ourselves sometimes.” (welfare professional)*

*Connective musical involvement, job satisfaction, inspiration and enthusiasm:* Managers at the residential care center appreciate the staff’s collective effort in supporting the residents’ participation in choir singing. According to the welfare staff, facilitating choir singing contributes to their own job satisfaction and energy. Team leaders in the hospital observe that musical performances of Bedside Music reduce irritability among staff. Welfare professionals at the residential care center are inspired by the choir members’ growth and enjoyment. They feel proud of the choir’s development, the conductor’s progress and the mutual support among residents.

*“A resident sang a solo, and others praised her. That kind of encouragement really helps people grow.” (welfare professional)*

#### 3.2.3 Interpretations and implications.

The thresholds experienced by professionals can be summarized as *professional differences*, while their connective experiences may be described as *professional enrichment.* Compared to the dynamics *within* the musical encounters, the threshold experiences at the professional level appear to be more difficult to overcome, and the experiences of connection harder to attain. Despite professionals’ overall positive attitude towards musical encounters in healthcare, this does not automatically translate into shared responsibility and willingness to get involved. Care professionals in particular focus on the wellbeing of patients or residents and therefore perceive the musical performances primarily from this perspective. At this level as well, threshold experiences do not seem to be a necessary condition for experiences of connection. When they occur, they may either obstruct or enrich them. The findings at this level, which focus on the professionals’ perspective on their own experiences, constitute the central contribution of this study.

### 3.3 Experiences within the broader organization

From an organizational perspective, the journey from initial idea to the realization of musical encounters and structural collaboration between care and the arts, is marked by both threshold and connective experiences (Table 5).

**Table 5:** Threshold and connective experiences within the broader organization.

Experiences of connection within the broader organization	Threshold experiences *(staying out)*	Connective experiences *(opportunities)*
	• unclear or unrealistic expectations • lack of shared responsibilities, involvement and commitment, or influence • short-term and problem-focused policymaking or lack of imagination • financial constraints or unexpected fuss.	• connection across positions, departments, organizations, or sectors • connection between professionals and their profession or employer • connection between present and future institutional challenges

#### 3.3.1 Organizational threshold experiences.

*Unclear or unrealistic expectations:* Between the conservatory students and care staff involved in the concert by and for colleagues, there was confusion about what they could expect from one another. Consequently, some of them became overburdened, disappointed, or lost their motivation. The project leaders at the residential care center and the center for arts and culture conclude that they had initially been overly enthusiastic.

*Lack of shared responsibility, involvement and commitment, or influence:* In the case of residential care center, frequent changes in coordination and a lack of commitment from the management of both collaborating institutions, disrupt the music project’s progress and erode mutual trust. Care managers observe that most care professionals have not fully engaged in the project. Likewise, according to their manager, arts teachers express little enthusiasm for working with elderly people in residential care settings.

In all cases, initiators initially lack the mandate needed to realize their idea for a music project. They overcome this hurdle by mobilizing someone who is committed to the idea and possesses the necessary influence to realize it. At the residential care center, project leaders eventually feel exhausted and disillusioned due to their limited range of influence.

*Short-term policymaking or lack of imagination:* Project leaders at the residential care center criticize the reactive nature of policymaking within the institution, which tends to focus on immediate problems rather than long-term opportunities. They argue that policymakers overlook the music project’s potential to address broader institutional challenges.

*“Everyone said, ‘Great idea!’… But when it comes to actually doing what’s needed, they didn’t show up.” (project leader)*

While the management team expresses interest in integrating music into personnel policy, it acknowledges struggling to envision meaningful ways of implementing it.

*Financial constraints or unexpected fuss:* Due to the loss of government funding, the residential care center’s board plans to require choir members to contribute financially to the choir rehearsals, a measure that team leaders expect will discourage participation among the singers. The center for arts and culture, which depends on municipal subsidies, feels particularly accountable for engaging independently living elderly, rather than those residing in care institutions. In the hospitals, budget shortages make structural funding for music initiatives unfeasible.

*“They would like to make it happen, but the resources just aren’t available.” (facilitator)*

As the music project at the residential care center progresses, competition between care locations generates some friction, while frequent leadership changes disrupt coordination at both the residential care center and the arts and culture center.

#### 3.3.2 Experiences of organizational connection.

*Connection across positions, departments, organizations, or sectors:* In the case of the concert by and for colleagues at the academic medical center, the facilitator, who is also a manager, can align the initiative with a broader organizational goal: promoting employee well-being. In this role, he is well-positioned to mobilize support and embed the concert within the organization.

As a member of the management team, the general hospital’s facilitator is well-positioned to pave the way for Bedside Music, by informing and enthusing the hospital’s managers and by securing a succession of annual contracts. The musicians of Bedside Music are gradually becoming familiar with the hospital, which makes them feel more at home, experience more independence, develop a sense of freedom and express their needs, such as a dressing room.

*“We simply take the space we need… Step by step, very kindly, very calmly, adding just a little bit more each time.” (musician)*

*Connection between employees and their profession or employer:* Facilitators of musical encounters are often deeply motivated by a combination of personal and professional motivation. For example, a former nurse views her facilitating role as an extension of the care she once provided to patients. A former musician finds pride and fulfillment in organizing a concert by and for colleagues at an academic medical center, an initiative that aligns with the medical center’s Human Resources-goals of strengthening employees’ connection to their work and institution.

*“When you see the nursing staff skipping through the corridor, you think: hat is what music does, hat’s what it brings! And that makes me happy; it simply makes me feel cheerful too“ (facilitator)*

*Connection to institutional challenges and future goals:* All facilitators view musical encounters as a chance to reduce the workload of care professionals and to give musicians the opportunity to contribute to healthcare. Project leaders in the case of the residential care center, believe that music can enhance person-centered care. They also see value in connecting the care and cultural sectors within the city, as this can support the continuity of cultural development for the older adults and help address loneliness in the aging population.

#### 3.3.3 Interpretations and implications.

At the organizational level, the threshold experiences could be summarized as *staying out* and the connective experiences as *opportunities* for the organization. Project leaders, care professionals and musicians generally perceive insufficiently consistent and sustained engagement from managers and board members in relation to music projects. They regard the opportunities at this level, particularly the opportunity to use music to address present and future institutional challenges, as both desirable and conceivable, yet too ambitious to attain. In contrast to the two levels discussed above, the threshold experiences at this level, characterized as management and board members *‘staying out*‘, may function both as a barriers to and preconditions for experiences of organizational connection.

The empirical evidence and implications of this interpretation is relatively limited, as the findings are primarily based on the experiences and interpretations of project leaders, care professionals and musicians, supplemented with observations of the researcher. Key actors at the organizational level were only minimally engaged in both the music project and the study. Nevertheless, these findings may provide valuable directions for both implementation and future research.

### 3.4 Overarching construct: precariousness

By abstracting the theme matrices (Tables 3–5), the overarching construct of *precariousness* emerged [33]. The term encompasses two dimensions: to be handled with care (*delicate*) and threatened by exclusion (*at risk*). These dual connotations reflect the core dynamics of musical encounters in healthcare that emerged from the analysis. The first, more positive, aligns with the delicate nature of musical connection, in which threshold experiences are inherent to the artistic process. The second, more negative connotation, relates to professional and organizational fragility, where thresholds often prove too difficult to overcome (Fig 1).

**Fig 1:**
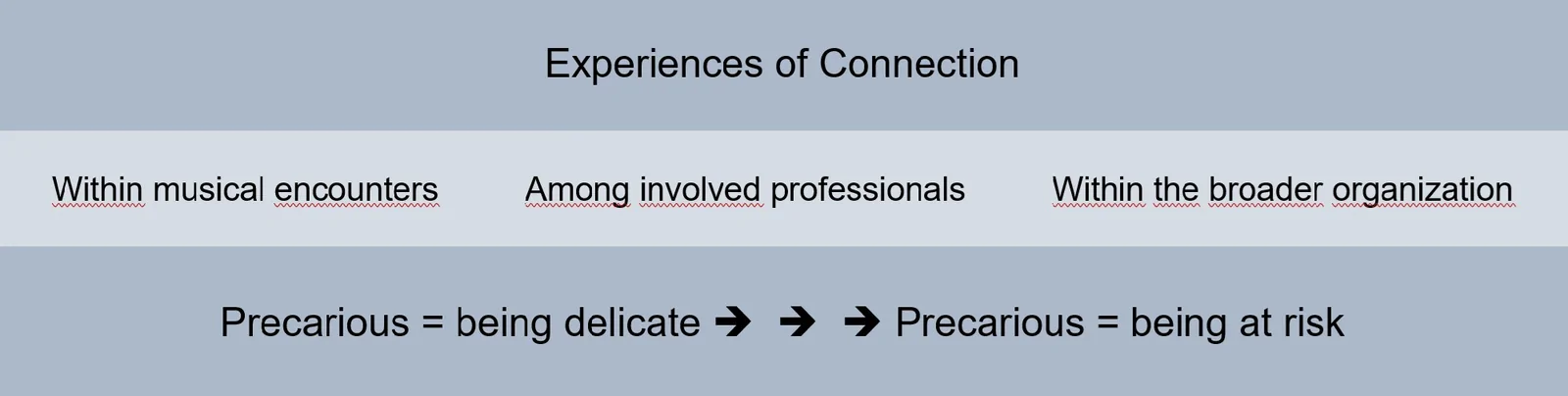
The precarious nature of musical encounters.

## 4. Discussion

### 4.1 Theory

The findings align with the scoping review of WHO-Europe, which highlights the health benefits of music, but notes challenges in translating evidence-based knowledge into practice [13]. Consistent with Clift [34], who identifies an attention deficit for wider issues and circumstances in the WHO-report, our study modestly suggests that (organizational) contexts play a critical role in enabling or obstructing musical encounters. Thresholds at the organizational level, such as short-term policymaking and limited managerial involvement and commitment, contrast with organizational opportunities like strategic positioning or employee retention, as well as with the potential for connective experiences of patients, residents and professionals. This hierarchy may suggest that sustainable implementation of musical encounters in healthcare requires attention to organizational culture and leadership.

The findings also may connect musical encounters to broader healthcare challenges. For instance, shared musical enjoyment and inspiration may support quality of care, job satisfaction and staff retention, while improved employee-employer relationships could inform more efficient and effective care structures. These remain hypothetical implications rather than outcomes demonstrated in the present study. However, recognizing the potential contribution of musical encounters to future institutional challenges may enable managers and board members to lead innovations more effectively. Furthermore, inter-organizational collaboration, if not hindered by unclear responsibilities and limited commitment, could further advance the role of arts in healthcare, as advocated by WHO Europe [13].

The theoretical view on human beings as inherently musical and caring [16,17] and the ethical and aesthetic perspective on healthcare [17,19,20] provide lenses through which the findings can be interpreted and reflected upon. Spitzer’s anthropological perspective on music making [16], and Tronto’s ethical-political perspective on the distribution of care responsibilities [17] are central to deepening the understanding of the observed dynamics. As participants’ aesthetic experiences were not explored in depth, the aesthetic perspectives of Thompson [19], De Kock [20] and Schiuma [21] on healthcare and its institutions, play a more supportive role, enriching and deepening the findings.

From an anthropological perspective, the findings affirm Spitzer’s view in *The Musical Human* that music resonates deeply with people, offering a wide range of connective experiences that are especially valuable in care contexts [16]. The artistry of professional musicians—through sensitivity and flexibility—enhances these experiences, supporting Spitzer’s claims about music’s capacity to evoke sensemaking, harmony, and inspiration. However, while participants conceptually acknowledge the potential of musical encounters to stimulate innovation, this was not demonstrated in practice in the study.

The experiences within the musical encounters also reflect Tronto’s conception of the caring human, which views care as inherently relational, recognizing that all individuals are both in need and capable of providing care [17]. This opens possibilities for musicians to be acknowledged as (artistic) caregivers. Yet, the study also highlights ethical and political dimensions of care, showing that hierarchy and voice may influence who is able to participate in, and shape, musical encounters as a form of care. For example, managers and board members often remained indifferent, while involved professionals and project leaders lacked authority.

Finally, the study may reinforce aesthetic perspectives on healthcare. Echoing Thompson [19] and De Kock et al. [20], the findings may indicate that musicians can enhance the quality of both care and care work, through evoking aesthetic sensations. These findings also may resonate with Schiuma’s [21] ideas about the value of aesthetic sensations in organization development, suggesting that similar principles could help care and cultural institutions navigate transitions. Despite participants acknowledging this potential, assumptions that musical encounters would benefit the organization or the wider healthcare system remained largely cognitive and aspirational in this study, rather than empirically realized.

As the literature suggests [35,36] musical encounters also have the potential to cause harm, as identified Music Induced Harm model [36]. In this study, minor incidents include a choir member fainting, a patient reacting with distress, and a care professional becoming nervous while preparing for a concert. Musicians faced risks such as exposure to illness, emotional rejection, and challenges to artistic freedom.

### 4.2 Methodology

This study faced several methodological limitations that shaped its scope and findings. First, in all three cases, some musical performances were cancelled or could not be observed due to COVID-19 measures. In addition, these restrictions prevented the researcher from interviewing patients/residents and care professionals who were not directly involved in the musical encounters. Secondly, care professionals, often working under time constraints, were generally less inclined to engage in the musical encounters, focusing instead on clinical responsibilities. Managers and board members were even more detached. As a result, their perspectives are underrepresented, limiting the completeness and depth of the findings.

It can be concluded that the exploratory nature of the study, the limited number of cases, and restricted access to events and participants due to the COVICD-19 measures, the transferability and broader implications of the findings are limited. However, the methodology’s strength lies in its contextual richness, revealing the importance of the organizational context surrounding the traditional tripartite relationship between patient, professional, and musician in musical encounters in healthcare. Moreover, the researcher’s familiarity with music, healthcare, and diverse perspectives enabled nuanced observation, interviewing, and interpretation.

## 5. Conclusions

This study reveals that musical encounters in healthcare settings may foster meaningful experiences of connection among individuals and within the professional and organizational context. However, these experiences emerge through a precarious process that must navigate various threshold experiences. Some thresholds are so low that they may remain unnoticed, while others are clearly evident. At times, threshold experiences are productive, fostering connective experiences (e.g., within musical encounters), sometimes even serving as a precondition for connection to occur. At other times, they prevent experiences of connection altogether. Consistent with the arts therapy literature, these dynamics are inherently personal, relational, and situational, and the how and why still remains unclear [37].

Based on the findings, it is recommended to recognize both the caring and musical nature of every human being in healthcare, for example, by systematically integrating musical encounters into healthcare settings and recognizing musicians’ dual roles as artist and caregiver. This approach could broaden access to their evidence-based benefits and possibly even may enhance the sustainability of the healthcare system. Expanding musical diversity, such as rotating artists in residence, can meet varied listener preferences. Education in music, care, and management should emphasize both theoretical and experiential learning that applies to musical encounters in healthcare practices.

Further research could explore new cases and methodological approaches to validate and extend these findings, particularly by incorporating contexts and perspectives that were underrepresented in the present study, including the direct voice of patients/residents, professionals who are not directly involved in musical activities, and the perspectives of managers and board members.

Finally, the concept of precariousness is proposed as a foundation for a validated framework to guide future research, collaboration, and education. Acknowledging the precarious nature of musical encounters can help position musicians as genuine partners in care and ensure their contributions are practically supported.
