# Supplementary material for: The precarious nature of musical encounters in healthcare: An ethnographic multi-case study focusing on the perspectives of care professionals and musicians
Source: PLoS One. 2026 Oct 5;21(10):e0359306. doi: 10.1371/journal.pone.0359306 (PMC13637854; doi:10.1371/journal.pone.0359306)
Supplement: Supplementary file 1 [file pone.0359306.s001.docx]

**Response to Academic Editor and Reviewers**

**Manuscript title:** The Precarious Nature of Musical Encounters in Healthcare: An Ethnographic Multi-Case study Focusing on the Perspectives of Care Professionals and Musicians (previous title: The Precarious Nature of Musical Encounters in Healthcare: An Ethnographic Multi-Case Study)
**Journal:** PLOS ONE
**Manuscript ID:** [PONE-D-26-13817] - [EMID:de7335860df2f2c9]

**Authors:** Monique Bussmann, Mieke Cardol, Marieke Groot, Chris Kuiper

Dear Editor, Assoc. Prof. Phakkharawat Sittiprapaporn, Ph.D,

Thank you for handling our manuscript *The Precarious Nature of Musical Encounters in Healthcare: An Ethnographic Multi-Case Study Focusing on the Perspectives of Care Professionals and Musicians*, PONE-D-26-13817 (previous title: *The Precarious Nature of Musical Encounters in Healthcare: An Ethnographic Multi-Case Study*), and for the Reviewers’ careful reading.

We have revised the manuscript to address all comments. The main changes involve:

- Clarifying the ethical and legal restrictions on sharing a de-identified data set, as well as the authorities responsible for imposing these restrictions;
- Clarifying the empirical dataset, consolidating themes and strengthening conceptual coherence within the findings;
- Moderating the claims regarding the scope of the findings and the study’s contribution.

A point-by-point response follows; all page and line numbers refer to the revised manuscript with tracked changes. For clarity, we handle the comments of every reviewer in order of the review and reproduce each comment followed by our response and a brief indication of where changes are made in the manuscript.

We have labeled the Comments as follows:

- Editorial Comment
- Reviewer #1: Comments 1.1-1.19
- Reviewer #2: Comments 2.1-2.3
- Reviewer #3: Comments 3.1-3.4
- Reviewer #4: Comment 4.1

Reviewers’ comments are shown in italic, our responses in plain text.

Given the Reviewers’ request for careful technical editing, which led to numerous minor revisions that may obscure the more substantive changes to the manuscript, we have prepared two additional revision documents for your convenience:

- The revised manuscript, showing the revised text fragments while excluding tracked changes and minor technical edits (Revised Manuscript New Texts Marked in Red).
- A schematic overview of all comments and our responses (Scheme Response).

Finally, we have added an attachment to this letter as part of our response to the Editorial Comment (Statement_REC-RUAS_M.Bussmann).

**1. Response to Academic Editor – Data availability and ethics prompt**

**Editorial Comment:**
*If there are ethical or legal restrictions on sharing a de-identified data set, please explain them in detail (e.g., data contain potentially identifying or sensitive patient information, data are owned by a third-party organization, etc.) and who has imposed them (e.g., a Research Ethics Committee or Institutional Review Board, etc.). Please also provide contact information for a data access committee, ethics committee, or other institutional body to which data requests may be sent.*

**Response:**
This Editorial Comment – and similar comments from reviewers – prompted us to reconsider how we described the ethical issues in the manuscript and to clarify these issues in the new manuscript, accentuating the ethnographical design of the study, the specific research conditions at that time, and the focus on the perspectives of professionals (musicians and care professionals), as reflected in the new, expanded title of the manuscript (see also Reviewer #4’ Comment 4.1). We discussed the revisions with the university’s data officers, who verified their advice with the administrators of the DataverseNL repository platform. Furthermore, we consulted the chair of the university’s Research Ethics Committee for advice, who provided a statement supporting the ethical procedures we followed (see Attachment ‘Statement_REC-RUAS_M.Bussmann’).

**Revisions:**

**p12**: ‘All data, including interviews, observations, reports, andforms, as well as all analysis steps were securely stored in SURF Research Drive. Metadata were published on the repository platform DataverseNL (<https://dataverse.nl/dataset.xhtml?persistentId=doi:10.34894/FBHF2Z>). In accordance with the platform’s decision tree (<https://zenodo.org/records/18390982>), the data were assessed and deemed ineligible for publication, therefore only the metadata were published.’

**p14**: ‘Although RUAS aims for research data to be publicly available wherever possible, according to the prevailing data protection and confidentiality requirements, researchers of the university are not permitted to publicly share full transcripts or field notes, because of its contextual richness which makes full de-identification impossible without severely compromising the scientific value of the dataset and the participants’ consent.’

**p14:** ‘The musical encounters were initiated as innovative activities by the participating care institutions, rather than as a study intervention. Consequently, the researcher did not intervene in the natural setting, which is consistent with the ethnographic research design. As no data were collected directly from patients or residents, and no personal or health-related data were gathered at all, the study did not require medical-ethical approval. At the time the research design was reviewed (2019), RUAS did not yet have a Research Ethics Committee. The secretary of the current committee can be approached with requests for data access (e-mail [eco@hr.nl)](mailto:eco@hr.nl)).’

**2. Response to Reviewer #1**

We thank Reviewer #1 for the detailed and thoughtful comments and for recognizing the potential contribution of our study. We respond to each point below.

**Formatting, Language, and Reference Issues**

**Comment (1.1):** The *manuscript requires careful technical editing before it can be considered for publication. There are numerous spelling, grammatical, formatting, and reference-list problems throughout the paper. While some of these issues may appear minor individually, their accumulation weakens the overall professionalism and credibility of the manuscript.*

**Response:** Prompted by the Reviewers’ comments we have improved the writing by addressing the Reviewers’ comments, by thoroughly rereading the text and making the necessary corrections, and finally having it edited by a native English speaker.’

**Comment (1.2):** *Several clear spelling and typographical errors should be corrected. For example, “review bord” should be “review board”; “Healtcare Settings” should be “Healthcare Settings”; “KNAW, 1018” appears to be a mistaken year and should presumably be “KNAW, 2018”; and the acknowledgement sentence “We like to thank...” should be revised to “We would like to thank...” or another standard formulation. There are also grammatical errors such as “Initial unease appear to play...” and “The findings also connects...”, both of which require correction.*

**Response:** We have addressed these specific issues and carefully checked the manuscript for other errors correcting those identified.

**Comment (1.3)**: *The manuscript also contains formatting inconsistencies, including irregular spacing around dashes, inconsistent punctuation, and a visible double-parenthesis error in “(Table 3)”. These issues should be addressed systematically rather than corrected only locally. Since the paper relies heavily on tables and thematic matrices, table formatting should also be checked carefully to ensure that all headings, categories, and subthemes are clear and consistently presented.*

**Response:** We have systematically addressed formatting inconsistencies in both the text and the tables.

**Comment (1.4):** *The reference list also needs thorough checking. Some entries appear incomplete, inconsistent, or potentially inaccurate. For example, one DOI appears to be repeated in a way that may not correspond to the cited article, and at least one reference seems only weakly connected to the claim it is used to support. The authors should verify every reference against the source text, ensure that author names, years, titles, journal details, page ranges, and DOIs are correct, and bring the reference list into full compliance with the journal’s style.*

**Response:**

- We have thoroughly checked both the reference list and all in-text references, making corrections, such as the DOI’s in question.
- We have checked the correspondence between the text and the cited sources and found that some links were indeed tenuous, although they could be justified because they are cited in the Scoping Review on Arts and Health [13]. To strengthen the manuscript and avoid overinterpretation, we decided to remove these references (Ayca et al., Crawford et al., Kaser et al., and Simpson et al.).
- We have incorporated some additional references, including Viola et al. [4], van de Berg et al. [6], Thompson [19], Fancourt et al. [28], Rosenberg [30], Tong et al. [31], and De Witte et al. [37]. Some of these were included to update and strengthen the manuscript, whereas others were added in response to Reviewers’ comments (discussed later in the response). Finally, we revised the reference list to conform to the Vancouver style required by PLOS One.

**Methodological Transparency and Qualitative Reporting Standards**

**Comment (1.5):** *However, the methods section does not yet provide enough detail about the empirical dataset. The manuscript states that the researcher used open observations, open interviews, reflection reports, and an elicitation technique based on an emotional process form. It also states that data were compiled into a single database and analyzed in Atlas.ti. However, the reader is not given sufficiently clear information about the number of interviews, number and type of participants, number of observations, duration of fieldwork, number of reflection reports, number of emotional process forms, or the distribution of these materials across the three cases.*

**Response:**

- To provide the requested details about the empirical dataset, we have made two revisions. First, we expanded Table 1 ‘Overview cases’ **(p9-10)** by adding a column that specifies the duration of each case. Second, we added Table 2 ‘Database: Cases and Data’ **(p11-12)** which provides detailed information on the participants and data for each case.
- To clarify the data analysis, we added the following information: ‘Within each case, data collection continued until no substantive new themes emerged in the observations and interviews, or until institutional access restrictions due to the COVID-19-pandemic made further fieldwork impossible.’ **(p11)**

And:

‘The data were compiled into a single database and qualitatively analyzed using Atlas.ti. The analysis followed an inductive process of constant comparison: open coding identified meaningful occurrences and participant interpretations, followed by axial coding, using the group code function in Atlas.ti.’ **(p12)**

**Comment (1.6):** ‘*This lack of detail makes it difficult to assess the depth and balance of the evidence. I recommend that the authors add a clear data overview table showing, for each case, the types of data collected, number of participants, participant roles, number of interviews, number of observations, and any supplementary materials used. This would significantly improve the transparency and credibility of the qualitative design. Since this is a qualitative ethnographic multiple-case study, the authors should also ensure that the manuscript fully follows appropriate qualitative reporting standards, including the COREQ checklist mentioned in the manuscript, particularly regarding participant characteristics, recruitment, data volume, researcher reflexivity, analytic procedures, and the adequacy of the empirical material.’*

**Response:** We refer to our response above **(Comment 1.5).**

We have reapplied the COREQ checklist [31] to ensure that the manuscript fully complies with qualitative reporting standards. Consequently, we have added:

- Table 2 ‘Database: Cases and Data’ **(p9-10),** describing participant characteristics and data volume;
- More details on participant recruitment **(p8).** ‘The individual participants (such as project leaders, care professionals and musicians) were recruited by the respective facilitators, provided with information about the study, and invited to participate voluntarily.’;
- ’More detailed information on the data collection and analytic procedures **(p11**): ‘Within each case, data collection continued until no substantive new themes emerged in several of the observations and interviews, or until institutional access restrictions due to the COVID-19-pandemic made further fieldwork impossible.’;
- A section Fieldwork **(p13)** describing the researcher’s reflexivity and the adequacy of the empirical material:

‘The study coincided with the COVID-19 pandemic, which demanded the full attention of healthcare institutions and made them highly reluctant to grant access to musicians and, even more, to researchers. Consequently, the researcher’s access for data collection was largely limited to musicians, project leaders/facilitators and directly involved staff members, rather than to patients/residents, care staff and managers.

As a result, the dataset does not include the direct voices of patients/residents and healthcare professionals who were not directly involved, or board members. However, the musicians, project leaders/facilitators, and professionals who participated directly proved both willing and able to represent the perspectives of patients/residents, other professionals, and managers. It can be concluded that this has limited ability to answer the research question with the desired comprehensiveness. Nevertheless, these specific circumstances provided particularly rich insights into the dynamics at the professional level, as well as at the other levels.’

**Comment (1.7):** *The authors should also clarify the role of the researcher in the field. The manuscript mentions that the researcher sometimes participated modestly, for example by singing or assisting participants, and at other times observed unobtrusively. This is appropriate for ethnographic work, but it requires more reflexive discussion. The authors should explain how the researcher’s professional background, existing networks, and occasional participation may have shaped access, observation, interpretation, and participant responses.*

**Response:** We refer to point 4 in our response to **Comment (1.6)** above, particularly the following text in the Fieldwork section regarding the researcher’s reflexivity **(p13-15):** ‘Being a former nurse and project manager in healthcare, as well as an amateur musician herself, the researcher is acquainted with the worlds of experience of respondents and capable of getting access, in-depth observation and interviewing, analyzing, and interpreting. As an experienced qualitative researcher, she continuously reflected on which attitude would best preserve the naturalness of the situation, and remained aware that, despite these efforts, her presence may have influenced the participants’ behavior.’ **(p13)**

**Ethics and Data Reporting**

**Comment (1.8):** *The ethics statement requires urgent clarification. The submission information states “N/A” for ethics, while the manuscript clearly involves human participants and observations in healthcare environments. Because PLOS ONE requires appropriate approval or a clear justification for studies involving human participants, including observational studies and studies using personal data, the ethics statement should not simply be marked as “N/A” without a more precise explanation. The manuscript explains that the study was not medical in nature and that the institutions involved, together with the university of applied sciences, determined that no medical IRB review was required. However, this explanation is not sufficient in its current form.’*

**Comment (1.9):** *The authors should clarify whether a formal ethics review exemption or waiver was obtained, which institution or body made this determination, and whether there is a documented decision. They should also explain more precisely how informed consent was obtained, especially for observations in healthcare settings and for any participants who were not directly interviewed. The manuscript states that verbal informed consent was captured in interview recordings, but it remains less clear how consent was handled for observed musical encounters involving patients, residents, visitors, or staff who may not have been interviewed.*

*This issue is especially important because the study takes place in healthcare and residential care environments, where questions of vulnerability, privacy, and consent are particularly sensitive. Even if the study is not a medical intervention, it still involves human participants and should therefore provide a more robust ethics statement.*

**Comment (1.10)** *The data availability statement also needs clarification. I understand that full qualitative data may not be publicly shareable because of participant privacy and the sensitivity of healthcare and residential care settings. However, the current statement should specify whether any controlled access to the underlying data is possible, which institution or committee is responsible for access requests, what criteria would be used to assess such requests, and who qualified researchers should contact. If no access to the underlying data is possible, this should be clearly justified in relation to participant consent, privacy protection, and institutional requirements. This clarification is important for ensuring compliance with PLOS ONE’s data availability expectations while also protecting participants’ confidentiality*.

**Response:** In response to these three Comments, we refer to our response to the **Editorial Comment** above.

**Analytical Structure and Presentation of Findings**

**Comment (1.11)** *However, the current results section is overly fragmented. The analysis includes 34 subthemes across two major themes and three levels. While this shows empirical richness, it also produces a catalogue-like structure. Some subthemes are described only briefly, and the relationship between them is not always sufficiently analyzed. The paper would benefit from consolidating overlapping subthemes and giving more interpretive attention to the most important mechanisms*.

**Response:** We have consolidated overlapping subthemes, as presented in the revised Table 3 ‘Experiences within musical encounters’**(p16),** revised Table 4 ‘Experiences among involved professionals’ **(p20-21),** and revised Table 5 ‘Experiences within the broader organization’ **(p25).**

Furthermore, we have given more attention to the most important mechanisms by adding a subsection entitled ‘Interpretations and Implications’ to each results subsection (resp. **p19-20**, **p23-24**, and **p28-29**).

**Comment (1.12):** *In particular, the authors should make clearer how threshold experiences relate to connective experiences. Are threshold experiences necessary preconditions for connection? Are they barriers that must be overcome? Do some thresholds generate connection while others prevent it? Are the dynamics different at the level of the musical encounter, professional collaboration, and organizational embedding? At present, these relationships are sometimes asserted rather than fully demonstrated.*

**Response:** We have addressed this comment as follows in the three subsections ‘Interpretations and Implications’ mentioned above (Response to **Comment 1.11**) and in the section Conclusions:

- Experiences within the musical encounters **(p20):** ‘Although uncomfortable feelings often precede connective sensations, they are not a necessary precondition. Connections frequently occur without a preceding threshold experience. At the same time, initial uncomfortable feelings can act as a barrier to connection; conversely, they may also enhance and deepen them. Within musical encounters, most listeners appear willing and motivated to overcome their initial uneasiness and to engage in connective experiences.’;
- Experiences among involved professionals **(p24**): ‘At this level as well, threshold experiences do not seem to be a necessary condition for experiences of connection. When they occur, they may either obstruct or enrich them. The findings at this level, which focus on the professionals’ perspective on their own experiences, constitute the central contribution of this study.’;
- Experiences within the broader organization **(p29):** ‘In contrast to the two levels discussed above, the threshold experiences at this level, characterized as management and board members *‘staying out’,* may function both as barriers to and preconditions for experiences of organizational connection. The empirical evidence and implications of this interpretation are relatively limited, as the findings are primarily based on the experiences and interpretations of project leaders, care professionals and musicians, supplemented with observations of the researcher. Key actors at the organizational level were only minimally engaged in both the music project and the study. Nevertheless, these findings may provide valuable directions for both implementation and future research.’;
- Conclusions **(p34):** ‘Some thresholds are so low that they may remain unnoticed, while others are clearly evident. At times, threshold experiences are productive, fostering connective experiences (e.g. within musical encounters), sometimes even serving as a precondition for connection to occur. At other times, they prevent experiences of connection altogether. Consistent with the arts therapy literature, these dynamics are inherently personal, relational, and situational, and the how and why still remains unclear [37].’

**Referring to Reviewer #1’s own academic thoughts (1.13):**We are very grateful to Reviewer #1 for providing additional suggestions to strengthen the conceptual coherence and readability of the manuscript, drawing on their own academic expertise. We gave these suggestions careful consideration, which is reflected in our responses to **Comments (1.11) and (1.12**).

**Theoretical Framing and Positioning within Arts-in-Health, Music Therapy, and Creative Arts Therapy Research**

**Comment (1.14):** *However, in its current form, the theoretical framework is overextended. The manuscript moves quickly between different bodies of literature, and not all of them are equally integrated into the empirical analysis. As a result, the reader may find it difficult to determine which theoretical concepts are central to the manuscript’s original contribution and which are mainly contextual or supportive.*

**Response:** We have added the following text to the Discussion **(p31):** ‘The theoretical view on human beings as inherently musical and caring [16, 18] and the ethical and aesthetic perspective on healthcare [18,19,20] provide lenses through which the findings can be interpreted and reflected upon. Spitzer’s anthropological perspective on music making [16], and Tronto’s ethical-political perspective on the distribution of care responsibilities [18] are central to deepening the understanding of the observed dynamics. As participants’ aesthetic experiences were not explored in depth, the aesthetic perspectives of Thompson [19], De Kock [20] and Schiuma [21] on healthcare and its institutions, play a more supportive role, enriching and deepening the findings.’

Furthermore, we have reduced the emphasis on the work of moral philosopher Walker in subsection ‘Ethical an aesthetic perspectives on healthcare’ **(p6)** and on feminist philosopher Butler in the subsection ‘Overarching construct: precariousness’ **(p29).**

**Comment (1.15): p9** *The authors may also consider using music therapy and arts therapy literature to refine the meaning of “threshold experiences.” In the current manuscript, threshold experiences include discomfort, embarrassment, uncertainty, unfamiliarity, role ambiguity, professional difference, work pressure, and organizational resistance. These are important findings, but they could be theorized more precisely. In music therapy and arts therapy, discomfort or vulnerability is not always a negative barrier; it can sometimes be part of the process through which emotional expression, trust, and connection become possible.*

**Comment (1.16): p9** *However, this is not always the case. Some thresholds may be artistically or relationally productive, while others may be institutionally obstructive. This distinction is central to the manuscript’s own argument about precariousness. At the level of the musical encounter, uncertainty and sensitivity may be part of the delicate artistic process. At the organizational level, however, lack of funding, unclear responsibility, weak managerial commitment, and short-term policymaking are less likely to be productive thresholds; rather, they place the practice at risk. The authors should make this distinction more explicit.*

**Response to both comments**: Here, we refer to our responses on **Comments (1.11) and (1.12**). Furthermore, we have added a reference to De Witte et al. [37] regarding the mechanisms of change in the creative arts therapies **(p34).**

**Scope of Claims and Contribution**

**Comment (1.17)** *The manuscript makes several important claims about the potential of musical encounters to support connection, professional enrichment, interprofessional collaboration, employee well-being, and organizational innovation. These are valuable directions. However, some claims should be moderated because the empirical material does not always fully support the broader implications. For example, the discussion suggests that musical encounters may contribute to staff retention, employee-employer relationships, and healthcare system sustainability. These are plausible implications, but they are not directly demonstrated by the data in the current version.*

**Response:** We agree with the reviewer and have added the following text to the Discussion **(p31):** ‘The findings also may connect musical encounters to broader healthcare challenges. For instance, shared musical enjoyment and inspiration may support quality of care, job satisfaction and staff retention, while improved employee-employer relationships could inform more efficient and effective care structures. These remain hypothetical implications rather than outcomes demonstrated in the present study. However, recognizing the potential contribution of musical encounters to future institutional challenges may enable managers and board members to lead innovations more effectively.’

Furthermore, we added nuance to previous assertions **(p32):** ‘Finally, the study may reinforce aesthetic perspectives on healthcare. Echoing Thompson [19] and De Kock et al. [20] the findings may indicate that musicians can enhance the quality of both care and care work, through evoking aesthetic sensations. These findings also may resonate with Schiuma’s [21] ideas about the value of aesthetic sensations in organization development, suggesting that similar principles could help care and cultural institutions navigate transitions. Despite participants acknowledging this potential, assumptions that musical encounters would benefit the organization or the wider healthcare system remained largely cognitive and aspirational in this study, rather than empirically realized.’

**Comment (1:18): p10** *The authors should distinguish more clearly between findings, interpretations, and implications. The strongest findings concern experiences of connection and threshold within specific musical encounters and professional contexts. Broader organizational or policy implications should be framed more cautiously, especially given the limited number of cases and the fact that patients, residents, uninvolved staff, and senior managers are not directly or systematically represented as participant groups.*

**Response:** Here, we refer here to our responses to **Comments (1.12) and (1.17).**

**Others – Use of AI Tools and Language Editing**

**Comment (1.19): p10** *The disclosure that Microsoft 365 Copilot was used for English grammar checking and word-count reduction is appropriate in principle. However, the manuscript still contains numerous grammatical, typographical, formatting, and reference errors. The manuscript still requires careful human proofreading and consistency checking.
Disclosure of Microsoft 365 Copilot use is appropriate, but manuscript still needs careful human proofreading.*

**Response:** Here, we refer hto our responses on **Comments (1.1 to 1.4) and (2.1 to 2.2).**

**General Appreciation**

**Reviewer comment:**
*The manuscript could make a meaningful contribution to research on arts in health, relational care, healthcare work environments, and transdisciplinary collaboration.*

**Response:**
We thank Reviewer #1 for these encouraging remarks. We hope that the revisions in language, methods, ethics, analysis, and theory strengthen the manuscript’s contribution in the ways suggested.

**3. Response to Reviewer #2**

We are grateful to Reviewer #2 for evaluating the manuscript as extremely valuable, well written, and suitable for publication, and for providing helpful suggestions for further improvement.

**Comment: (2.1):** *In the Discussion, some conceptual passages concerning “connection” and “precariousness” could be slightly streamlined to avoid occasional repetition.*

**Response***:* We thoroughly revised the Discussion **(p30-34),** in response to several comments. In the revised version we avoided repetition of key concepts.

**Comment (2.2): p11** *A final careful language editing by a native English speaker or professional editor may help improve fluency in a few passages, although the manuscript is already fully intelligible and generally written in good academic English.*

**Response:** Here, we refer here to our response to Reviewer #1’s **Comment (1.1)** above.

**Comment (2.3): p11** *Although the methodological limitations are acknowledged appropriately, the manuscript could further clarify the implications of the restricted access to certain participant groups (patients, uninvolved staff, administrators) for the transferability of the findings*.

**Response:** Here, we refer here to our response to Reviewer #4’s **Comment (4.1)** below.

**4. Response to Reviewer #3**

We thank Reviewer #3 for the careful reading and for highlighting important issues regarding missing direct voices, citation errors and ethical ambiguities. We have reviewed it carefully and hope that we have been able to address any concerns.

**Major Revisions**

**Comment (3.1) *titled ‘Critical Citation Mismatch’****:* *In lines 150-151, the authors state that musical encounters foster deep and multifaceted experiences of connection, citing "Simpson et al. [28]" as a reference. However, Reference 28 in the reference list is a medical meta-analysis titled "A meta-analysis of the association between adherence to drug therapy and mortality." This is an entirely unrelated medical paper that has no relevance to music or arts in health. The authors must rigorously audit their entire manuscript and correct this severe error.*

**Response:** Here, we refer to our responses to **Comments (1.1 to 1.4),** particularly **Comment (1.4),** which discusses the reference to Simpson et al.

**Comment (3.2) *titled ‘Data Availability Policy Non-Compliance’****. PLOS ONE maintains a strict data availability policy. The authors have stated that the data are not publicly available to protect participant privacy and have only provided metadata on DataverseNL. Under PLOS ONE guidelines, if raw data cannot be shared publicly, the authors MUST provide a non-author, independent institutional contact (e.g., a research ethics committee or data access committee) to which future researchers can apply for data access. Relying solely on metadata or a personal repository link is insufficient.*

**Response:** Here, we refer to our response to the **Editorial Comment** above.

**Comment (3.3) titled ‘*Ethics and Consent Clarification’****. The authors state that the study required no ethical review (IRB) because it is non-medical. However, since the study involves human participants undergoing observations and interviews, a formal institutional waiver or exemption status should be verified. Please clarify whether a formal exemption was officially granted by the university's ethics board. Furthermore, please justify why verbal informed consent was preferred over written consent, and explain how the voluntariness of vulnerable populations (e.g., patients in general hospitals or residents in care homes) was protected.*

**Response***:* In addition to our response to the **Editorial Comment** we have provided following extra information in the revised manuscript.

- Referring to the section ‘Ethical Considerations’ **(p13-15),** we have added: The musical encounters were initiated as innovative activities by the participating institutions, rather than as a study intervention. Consequently, the researcher did not intervene in the natural setting, which is consistent with the ethnographic research design.’ As well as the sentence: ‘All verbal informed consents were captured in the recordings of the interviews, that in most cases were conducted online due to COVID-19-measures.’;
- To clarify the impact of COVID-19 restrictions on the research process (e.g., the inability to conduct face-to-face interviews and obtain written consent), we have referred to these restrictions more explicit throughout our revision process.;
- Referring to the Data Collection, we have added the following information **(p11**): In cases where COVID-19 restrictions prevented observation, participating musicians, welfare and care professionals submitted reflection reports.’ As well as the sentence: ‘Within each case, data collection continued until no substantively new themes emerged in the observations and interviews, or until institutional access restrictions due to the COVID-19-pandemic made further fieldwork impossible.’;
- Referring to Ethical Considerations (**p13-15)**, we have added: ‘…*that in most cases were conducted online due to COVID-19-measures.’ to the sentence ‘All verbal informed consents were captured in the recordings of the interviews’;*
- Finally, referring to the methodological discussion **(p33-34)** we added following information: In all three cases, some musical performances were cancelled or could not be observed due to COVID-19 measures. In addition, these restrictions prevented the researcher from interviewing patients/residents and care professionals who were not directly involved in the musical encounters.’

**Minor Revisions**

**Comment (3.4):** *The authors noted that they were restricted to interviewing only the professionals involved, thereby omitting the direct voices of patients, residents, and uninvolved staff. This limitation should be integrated more deeply into the Discussion, reflecting on how the absence of these core perspectives might constrain the completeness of the "Connective experiences" matrix.*

**Response:** Addressing this revision point:

- In the new section ‘Fieldwork’ **(p13)** we added the following phrase: ‘The study coincided with the COVID-19 pandemic, which demanded the full attention of healthcare institutions and made them highly reluctant to grant access to musicians and, even more, to researchers. Consequently, the researcher’s access for data collection was largely limited to musicians, project leaders/facilitators and directly involved staff members, rather than to patients/residents, care staff and managers. As a result, the dataset does not include the direct voices of patients/residents and healthcare professionals who were not directly involved, or board members. However, the musicians, project leaders/facilitators, and professionals who participated directly proved both willing and able to represent the perspectives of patients/residents, other professionals, and managers. It can be concluded that this has limited ability to answer the research question with the desired comprehensiveness. Nevertheless, these specific circumstances provided particularly rich insights into the dynamics at the professional level, as well as at the other levels.’;
- In the subsection Methodological discussion **(p33)** we added the following text: ‘First, in all three cases, some musical performances were cancelled or could not be observed due to COVID-19 measures. In addition, these restrictions prevented the researcher from interviewing patients/residents and care professionals who were not directly involved in the musical encounters. Secondly, care professionals, often working under time constraints, were generally less inclined to engage in the musical encounters, focusing instead on clinical responsibilities. Managers and board members were even more detached. As a result, their perspectives are underrepresented, limiting the completeness and depth of the findings.’

**Comment (3.5):** *‘Typographical Error in References’. In Reference 30 (line 584), the publication year for the Netherlands Code of Conduct for Research Integrity is incorrectly printed as "1018" instead of "2018." Please correct this.*

**Response:** We have corrected this.

**5. Response to Reviewer #4**

We thank Reviewer #4 for highlighting the important issue of patient participation and the scope of the study.

**Comment (4.1):** *Without active participation of the patients through interview for data gathering, there is a gap regarding determination of the depth of this musical encounter and whether it fosters connection among the patients or with the care giver and musician. (…) If* patients experience cannot be ascertained; this should be reflected at the onset through a review of the title, abstract and the research question(s).

Because we largely agree with this comment, we have:

- Adjusted the title of the manuscript **(p1):** ‘The precarious nature of musical encounters in healthcare. An ethnographic multi-case study focusing on the perspectives of care professionals and musicians, including the abstract and research question.’;
- Reflected on this limitation in subsection ‘Interpretations and implications’ **(p20):** ‘An empirical limitation is the absence of patients’ and residents’ direct voices, since the findings are based on the musicians’ and care professionals’ experiences and observations regarding musical performances, supplemented by the researcher’s observations. Nevertheless, this limitation is somewhat offset by the wealth of existing studies that have already documented patients’ and residents’ perspectives on the role and effects of music in care settings [1,2,3,4]’.
- Discussed this limitation in the Methodological Discussion **(p30-34):**

‘This study faced several methodological limitations that shaped its scope and findings. First, in all three cases, some musical performances were cancelled or could not be observed due to COVID-19 measures. In addition, these restrictions prevented the researcher from interviewing patients/residents and care professionals who were not directly involved in the musical encounters. Secondly, care professionals, often working under time constraints, were generally less inclined to engage in the musical encounters, focusing instead on clinical responsibilities. Managers and board members were even more detached. As a result, their perspectives are underrepresented, limiting the completeness and depth of the findings.

It can be concluded that the exploratory nature of the study, the limited number of cases, and restricted access to events and participants due to the COVID-19 measures, the transferability and broader implications of the findings are limited. However, the methodology’s strength lies in its contextual richness, revealing the importance of the organizational context surrounding the traditional tripartite relationship between patient, professional, and musician in musical encounters in healthcare.’;

- incorporated it in the recommendations for further research in the Conclusions **(p35):** ‘Further research could explore new cases and methodological approaches to validate and extend these findings, particularly by incorporating contexts and perspectives that were underrepresented in the present study, including the direct voice of patients/residents, care professionals who are not directly involved in musical activities, and the perspectives of managers and board members.’

**6. Closing Statement**

We hope these revisions resolve the Editor’s and Reviewers’ concerns and look forward to your decision.

Sincerely,

Monique B.M. Bussmann, on behalf of all co-authors
