# Supplementary material for: The precarious nature of musical encounters in healthcare: An ethnographic multi-case study focusing on the perspectives of care professionals and musicians
Source: PLoS One. 2026 Oct 5;21(10):e0359306. doi: 10.1371/journal.pone.0359306 (PMC13637854; doi:10.1371/journal.pone.0359306)
Supplement: Peer review history

# Peer review: Decision Letter 0

**DOI:** 10.1371/journal.pone.0359306.r001

**Authors:** Assoc. Prof. Phakkharawat Sittiprapaporn

15 Jun 2026

Dear Dr. Bussmann,

Thank you for submitting your manuscript to PLOS ONE. After careful consideration, we feel that it has merit but does not fully meet PLOS ONE’s publication criteria as it currently stands. Therefore, we invite you to submit a revised version of the manuscript that addresses the points raised during the review process.

Please submit your revised manuscript by Jul 30 2026 11:59PM. If you will need more time than this to complete your revisions, please reply to this message or contact the journal office at plosone@plos.org. When you're ready to submit your revision, log on to https://www.editorialmanager.com/pone/ and select the 'Submissions Needing Revision' folder to locate your manuscript file.

- A letter that responds to each point raised by the academic editor and reviewer(s). You should upload this letter as a separate file labeled 'Response to Reviewers'.
- A marked-up copy of your manuscript that highlights changes made to the original version. You should upload this as a separate file labeled 'Revised Manuscript with Track Changes'.
- An unmarked version of your revised paper without tracked changes. You should upload this as a separate file labeled 'Manuscript'.

If you would like to make changes to your financial disclosure, please include your updated statement in your cover letter. Guidelines for resubmitting your figure files are available below the reviewer comments at the end of this letter.

If applicable, we recommend that you deposit your laboratory protocols in protocols.io to enhance the reproducibility of your results. Protocols.io assigns your protocol its own identifier (DOI) so that it can be cited independently in the future. For instructions see: https://journals.plos.org/plosone/s/submission-guidelines#loc-laboratory-protocols. Additionally, PLOS ONE offers an option for publishing peer-reviewed Lab Protocol articles, which describe protocols hosted on protocols.io. Read more information on sharing protocols at https://plos.org/protocols?utm_medium=editorial-email&utm_source=authorletters&utm_campaign=protocols.

As the corresponding author, your ORCID iD is verified in the submission system and will appear in the published article. PLOS supports the use of ORCID, and we encourage all coauthors to register for an ORCID iD and use it as well. Please encourage your coauthors to verify their ORCID iD within the submission system before final acceptance, as unverified ORCID iDs will not appear in the published article. *Only*  the individual author can complete the verification step; PLOS staff *cannot*  verify ORCID iDs on behalf of authors.

We look forward to receiving your revised manuscript.

Kind regards,

Assoc. Prof. Phakkharawat Sittiprapaporn, Ph.D.

Academic Editor

PLOS One

Journal Requirements:

When submitting your revision, we need you to address these additional requirements.

1. Please ensure that your manuscript meets PLOS ONE's style requirements, including those for file naming. The PLOS ONE style templates can be found at

https://journals.plos.org/plosone/s/file?id=wjVg/PLOSOne_formatting_sample_main_body.pdf and

https://journals.plos.org/plosone/s/file?id=ba62/PLOSOne_formatting_sample_title_authors_affiliations.pdf

2.  We note that you have indicated that there are restrictions to data sharing for this study. PLOS only allows data to be available upon request if there are legal or ethical restrictions on sharing data publicly. For more information on unacceptable data access restrictions, please see http://journals.plos.org/plosone/s/data-availability#loc-unacceptable-data-access-restrictions.

Before we proceed with your manuscript, please address the following prompts:

a) If there are ethical or legal restrictions on sharing a de-identified data set, please explain them in detail (e.g., data contain potentially identifying or sensitive patient information, data are owned by a third-party organization, etc.) and who has imposed them (e.g., a Research Ethics Committee or Institutional Review Board, etc.). Please also provide contact information for a data access committee, ethics committee, or other institutional body to which data requests may be sent.

b) If there are no restrictions, please upload the minimal anonymized data set necessary to replicate your study findings to a stable, public repository and provide us with the relevant URLs, DOIs, or accession numbers. For a list of recommended repositories, please see

https://journals.plos.org/plosone/s/recommended-repositories. You also have the option of uploading the data as Supporting Information files, but we would recommend depositing data directly to a data repository if possible.

We will update your Data Availability statement on your behalf to reflect the information you provide.

3. If the reviewer comments include a recommendation to cite specific previously published works, please review and evaluate these publications to determine whether they are relevant and should be cited. There is no requirement to cite these works unless the editor has indicated otherwise.

Additional Editor Comments :

Reviewers' comments:

Reviewer's Responses to Questions

**Comments to the Author**

1. Is the manuscript technically sound, and do the data support the conclusions?

Reviewer #1: Partly

Reviewer #2: Yes

Reviewer #3: Partly

Reviewer #4: Partly

**********

2. Has the statistical analysis been performed appropriately and rigorously?

Reviewer #1: N/A

Reviewer #2: Yes

Reviewer #3: N/A

Reviewer #4: N/A

**********

3. Have the authors made all data underlying the findings in their manuscript fully available?

The PLOS Data policy

Reviewer #1: No

Reviewer #2: Yes

Reviewer #3: No

Reviewer #4: Yes

**********

4. Is the manuscript presented in an intelligible fashion and written in standard English?

Reviewer #1: Yes

Reviewer #2: Yes

Reviewer #3: No

Reviewer #4: Yes

**********

Reviewer #1: Overall Review and Recommendation of The precarious nature of musical encounters in healthcare: An ethnographic multi-case study

I recommend major revision. My concerns are primarily about clarity, transparency, and reporting quality rather than the overall relevance of the topic. The manuscript addresses a meaningful and timely topic and has clear potential to make a useful contribution to the field of arts in health, particularly by examining live musical encounters not only as experiences for patients or residents, but also as relational, professional, and organizational practices. I do not recommend outright rejection, because the study has a promising empirical basis and an interesting central argument. However, I would not recommend acceptance in its current form. Substantial revision is required, especially in relation to formatting and reference accuracy, methodological transparency, ethics reporting, analytical integration, and theoretical clarification, as several issues currently affect the manuscript’s scholarly reliability, readability, and compliance with expected standards for qualitative research involving human participants.

1.Formatting, Language, and Reference Issues

The manuscript requires careful technical editing before it can be considered for publication. There are numerous spelling, grammatical, formatting, and reference-list problems throughout the paper. While some of these issues may appear minor individually, their accumulation weakens the overall professionalism and credibility of the manuscript.

Several clear spelling and typographical errors should be corrected. For example, “review bord” should be “review board”; “Healtcare Settings” should be “Healthcare Settings”; “KNAW, 1018” appears to be a mistaken year and should presumably be “KNAW, 2018”; and the acknowledgement sentence “We like to thank...” should be revised to “We would like to thank...” or another standard formulation. There are also grammatical errors such as “Initial unease appear to play...” and “The findings also connects...”, both of which require correction.

The manuscript also contains formatting inconsistencies, including irregular spacing around dashes, inconsistent punctuation, and a visible double-parenthesis error in “((Table 3)”. These issues should be addressed systematically rather than corrected only locally. Since the paper relies heavily on tables and thematic matrices, table formatting should also be checked carefully to ensure that all headings, categories, and subthemes are clear and consistently presented.

The reference list also needs thorough checking. Some entries appear incomplete, inconsistent, or potentially inaccurate. For example, one DOI appears to be repeated in a way that may not correspond to the cited article, and at least one reference seems only weakly connected to the claim it is used to support. The authors should verify every reference against the source text, ensure that author names, years, titles, journal details, page ranges, and DOIs are correct, and bring the reference list into full compliance with the journal’s style.

These technical issues are not merely cosmetic. In a qualitative article that makes a conceptual contribution, clarity and precision are essential. The current level of language and reference inconsistency may distract readers from the paper’s substantive argument and may also raise concerns about the care taken in preparing the manuscript.

2.Methodological Transparency and Qualitative Reporting Standards

The study adopts an ethnographic multiple-case design, which is appropriate for examining musical encounters in real-life healthcare settings. The three cases are potentially valuable: bedside cello performances in a general hospital, music-related activities in an academic medical center, and choir singing in residential care in collaboration with an arts and culture organization. This range of cases allows the authors to examine musical encounters across different settings, participant groups, and organizational arrangements.

However, the methods section does not yet provide enough detail about the empirical dataset. The manuscript states that the researcher used open observations, open interviews, reflection reports, and an elicitation technique based on an emotional process form. It also states that data were compiled into a single database and analyzed in Atlas.ti. However, the reader is not given sufficiently clear information about the number of interviews, number and type of participants, number of observations, duration of fieldwork, number of reflection reports, number of emotional process forms, or the distribution of these materials across the three cases.

This lack of detail makes it difficult to assess the depth and balance of the evidence. I recommend that the authors add a clear data overview table showing, for each case, the types of data collected, number of participants, participant roles, number of interviews, number of observations, and any supplementary materials used. This would significantly improve the transparency and credibility of the qualitative design. Since this is a qualitative ethnographic multiple-case study, the authors should also ensure that the manuscript fully follows appropriate qualitative reporting standards, including the COREQ checklist mentioned in the manuscript, particularly regarding participant characteristics, recruitment, data volume, researcher reflexivity, analytic procedures, and the adequacy of the empirical material.

The authors should also clarify the role of the researcher in the field. The manuscript mentions that the researcher sometimes participated modestly, for example by singing or assisting participants, and at other times observed unobtrusively. This is appropriate for ethnographic work, but it requires more reflexive discussion. The authors should explain how the researcher’s professional background, existing networks, and occasional participation may have shaped access, observation, interpretation, and participant responses.

3.Ethics and Data Reporting

The ethics statement requires urgent clarification. The submission information states “N/A” for ethics, while the manuscript clearly involves human participants and observations in healthcare environments. Because PLOS ONE requires appropriate approval or a clear justification for studies involving human participants, including observational studies and studies using personal data, the ethics statement should not simply be marked as “N/A” without a more precise explanation. The manuscript explains that the study was not medical in nature and that the institutions involved, together with the university of applied sciences, determined that no medical IRB review was required. However, this explanation is not sufficient in its current form.

The authors should clarify whether a formal ethics review exemption or waiver was obtained, which institution or body made this determination, and whether there is a documented decision. They should also explain more precisely how informed consent was obtained, especially for observations in healthcare settings and for any participants who were not directly interviewed. The manuscript states that verbal informed consent was captured in interview recordings, but it remains less clear how consent was handled for observed musical encounters involving patients, residents, visitors, or staff who may not have been interviewed.

This issue is especially important because the study takes place in healthcare and residential care environments, where questions of vulnerability, privacy, and consent are particularly sensitive. Even if the study is not a medical intervention, it still involves human participants and should therefore provide a more robust ethics statement.

The data availability statement also needs clarification. I understand that full qualitative data may not be publicly shareable because of participant privacy and the sensitivity of healthcare and residential care settings. However, the current statement should specify whether any controlled access to the underlying data is possible, which institution or committee is responsible for access requests, what criteria would be used to assess such requests, and whom qualified researchers should contact. If no access to the underlying data is possible, this should be clearly justified in relation to participant consent, privacy protection, and institutional requirements. This clarification is important for ensuring compliance with PLOS ONE’s data availability expectations while also protecting participants’ confidentiality.

4.Analytical Structure and Presentation of Findings

The manuscript’s central analytical distinction between threshold experiences and connective experiences is promising. It usefully avoids a simplistic account in which music is assumed to be automatically beneficial. Instead, the authors show that connection often emerges through discomfort, uncertainty, role ambiguity, professional differences, work pressure, and organizational obstacles. This is one of the strongest aspects of the manuscript.

However, the current results section is overly fragmented. The analysis includes 34 subthemes across two major themes and three levels. While this shows empirical richness, it also produces a catalogue-like structure. Some subthemes are described only briefly, and the relationship between them is not always sufficiently analyzed. The paper would benefit from consolidating overlapping subthemes and giving more interpretive attention to the most important mechanisms.

In particular, the authors should make clearer how threshold experiences relate to connective experiences. Are threshold experiences necessary preconditions for connection? Are they barriers that must be overcome? Do some thresholds generate connection while others prevent it? Are the dynamics different at the level of the musical encounter, professional collaboration, and organizational embedding? At present, these relationships are sometimes asserted rather than fully demonstrated.

4.1 Consolidating the 34 Subthemes and Strengthening Conceptual Coherence

Regarding the presentation of the 34 subthemes, I would like to emphasize that the following comment reflects my own academic reading of the manuscript rather than a necessary requirement that the authors must follow. The current thematic structure is understandable and clearly reflects the richness of the empirical material. Therefore, I do not suggest that the authors abandon their existing analytical framework. Rather, I offer this as a possible way to strengthen the manuscript’s conceptual coherence and readability.

At present, the manuscript organizes the findings into two major themes, namely threshold experiences and connective experiences, across three analytical levels: within musical encounters, among involved professionals, and within the broader organization. This is a promising structure. However, the further subdivision into 34 subthemes makes the results section appear somewhat dispersed. While the number of subthemes demonstrates the empirical richness of the ethnographic material, it also risks making the analysis read more like a catalogue of observed issues than a tightly developed argument. Some subthemes are highly relevant to the central claim of the paper, whereas others appear relatively descriptive or overlapping. As a result, the reader may find it difficult to identify which findings are analytically central and how the subthemes collectively support the overarching concept of precariousness.

One possible revision would be to consolidate some of the subthemes into a smaller number of higher-order analytical categories. For example, several threshold experiences within professional collaboration, such as differences in personality, role perception, musical preferences, embarrassment, concerns, and work pressure, could potentially be grouped under broader mechanisms such as “professional boundary negotiation,” “role uncertainty,” or “conditions of professional readiness.” Similarly, organizational-level issues such as unclear expectations, lack of shared responsibility, lack of commitment, lack of influence, financial constraints, and short-term policymaking might be integrated into a broader category such as “organizational embedding and institutional support.” This would allow the authors to preserve the empirical detail while presenting the findings in a more analytically concentrated form.

The manuscript would be stronger if it explained more explicitly how different threshold experiences function. Are they necessary transitional moments before connection can emerge? Are they barriers that need to be overcome? Are some thresholds productive because they open space for vulnerability, while others are obstructive because they reflect organizational neglect or lack of support? This distinction seems especially important for the paper’s central concept of precariousness, since precariousness is presented as both positive delicacy and negative vulnerability.

In this sense, I would encourage the authors to move from a mainly thematic presentation toward a more mechanism-based analysis. The current structure shows what kinds of threshold and connective experiences occurred. A revised structure could go further by explaining how these experiences interact. For instance, at the level of the musical encounter, discomfort, unfamiliarity, and sensitivity may be part of the artistic process through which connection becomes possible. At the professional level, differences in role perception or musical confidence may require negotiation before professional enrichment can occur.

Therefore, my suggestion is that the authors consider simplifying or reorganizing the 34 subthemes, not because the empirical material is excessive, but because the current presentation may obscure the manuscript’s central contribution. A more selective and integrated structure would help readers see more clearly how musical encounters move, or fail to move, from threshold experiences to connective experiences, and why this movement is best understood through the concept of precariousness. This revision would likely make the article more persuasive, more readable, and more theoretically impactful.

5. Theoretical Framing and Positioning within Arts-in-Health, Music Therapy, and Creative Arts Therapies Research

The following comments are intended not as a request for a substantially expanded theoretical review, but as suggestions for helping the authors clarify the hierarchy, function, and empirical relevance of the theoretical concepts already introduced in the manuscript.

The manuscript draws on several theoretical perspectives, including Spitzer’s view of the musical human, Tronto’s care ethics, Walker’s moral philosophy, aesthetic perspectives on healthcare, Schiuma’s work on arts in business, and Butler’s concept of precariousness. These perspectives are all relevant to the topic, and they demonstrate the authors’ ambition to frame musical encounters not merely as pleasant artistic additions to healthcare, but as relational, ethical, aesthetic, and organizational practices. This is a valuable direction. However, in its current form, the theoretical framework is overextended. The manuscript moves quickly between different bodies of literature, and not all of them are equally integrated into the empirical analysis. As a result, the reader may find it difficult to determine which theoretical concepts are central to the manuscript’s original contribution and which are mainly contextual or supportive.

The authors may also consider using music therapy and arts therapy literature to refine the meaning of “threshold experiences.” In the current manuscript, threshold experiences include discomfort, embarrassment, uncertainty, unfamiliarity, role ambiguity, professional difference, work pressure, and organizational resistance. These are important findings, but they could be theorized more precisely. In music therapy and arts therapy, discomfort or vulnerability is not always a negative barrier; it can sometimes be part of the process through which emotional expression, trust, and connection become possible.

However, this is not always the case. Some thresholds may be artistically or relationally productive, while others may be institutionally obstructive. This distinction is central to the manuscript’s own argument about precariousness. At the level of the musical encounter, uncertainty and sensitivity may be part of the delicate artistic process. At the organizational level, however, lack of funding, unclear responsibility, weak managerial commitment, and short-term policymaking are less likely to be productive thresholds; rather, they place the practice at risk. The authors should make this distinction more explicit.

6. Scope of Claims and Contribution

The manuscript makes several important claims about the potential of musical encounters to support connection, professional enrichment, interprofessional collaboration, employee well-being, and organizational innovation. These are valuable directions. However, some claims should be moderated because the empirical material does not always fully support the broader implications. For example, the discussion suggests that musical encounters may contribute to staff retention, employee-employer relationships, and healthcare system sustainability. These are plausible implications, but they are not directly demonstrated by the data in the current version.

The authors should distinguish more clearly between findings, interpretations, and implications. The strongest findings concern experiences of connection and threshold within specific musical encounters and professional contexts. Broader organizational or policy implications should be framed more cautiously, especially given the limited number of cases and the fact that patients, residents, uninvolved staff, and senior managers are not directly or systematically represented as participant groups.

7.Others

The disclosure that Microsoft 365 Copilot was used for English grammar checking and word-count reduction is appropriate in principle. However, the manuscript still contains numerous grammatical, typographical, formatting, and reference errors. The manuscript still requires careful human proofreading and consistency checking.

8.Conclusion

In conclusion, this is a promising manuscript with a worthwhile topic, an appropriate qualitative design, and a potentially valuable conceptual contribution. Its strongest contribution lies in showing that musical encounters in healthcare are not simply positive interventions, but precarious relational practices that depend on delicate artistic responsiveness and vulnerable organizational conditions.

However, the manuscript requires major revision. The authors should first address technical issues in formatting, language, and references. They should then substantially improve methodological transparency, clarify ethics and consent procedures, consolidate the thematic structure, strengthen the theoretical use of precariousness, and moderate broader claims that go beyond the evidence. If these issues are addressed carefully, the manuscript could make a meaningful contribution to research on arts in health, relational care, healthcare work environments, and transdisciplinary collaboration.

Reviewer #2: The manuscript presents an original, theoretically ambitious, and methodologically rigorous contribution to the growing field of arts in health. The study addresses a highly relevant and contemporary topic — the role of musical encounters in healthcare settings — moving beyond the more traditional understanding of music as a merely therapeutic or auditory stimulus and instead exploring its relational, organizational, ethical, and aesthetic dimensions. This broader perspective represents one of the major strengths of the article.

The ethnographic multi-case design is particularly effective and provides rich, nuanced, and convincing empirical material. The three selected cases allow the authors to explore musical encounters across different healthcare contexts and organizational settings, while maintaining a coherent analytical framework. The qualitative methodology is clearly described and appears appropriate to the research question. The thematic organization of the findings is especially strong, and the distinction between “threshold experiences” and “connective experiences” across the three analytical levels (musical encounters, professionals, organizations) offers a highly effective interpretative structure.

The article is also theoretically sophisticated. The dialogue established between music studies, care ethics, organizational theory, aesthetics, and healthcare humanities is impressive and handled with considerable intellectual maturity. In particular, the use of Judith Butler’s concept of precariousness as an overarching interpretative framework is insightful and original, allowing the authors to avoid simplistic or celebratory narratives about music in healthcare. The manuscript acknowledges tensions, fragilities, resistance, organizational limitations, and even possible forms of harm, thereby strengthening its scientific credibility.

Another important merit of the paper is its capacity to connect micro-level ethnographic observations with broader systemic questions concerning sustainability, workforce pressure, organizational culture, and transdisciplinary collaboration in healthcare. The study therefore contributes not only to arts-in-health scholarship, but also to wider debates on care, professional well-being, and institutional innovation.

Overall, I found the manuscript extremely valuable, well written, and suitable for publication in PLOS ONE.

I only have a few minor suggestions for improvement:

In the Discussion section, some conceptual passages concerning “connection” and “precariousness” could be slightly streamlined to avoid occasional repetition.

Although the methodological limitations are acknowledged appropriately, the manuscript could further clarify the implications of the restricted access to certain participant groups (patients, uninvolved staff, administrators) for the transferability of the findings.

A final careful language editing by a native English speaker or professional editor may help improve fluency in a few passages, although the manuscript is already fully intelligible and generally written in good academic English.

These are, however, relatively minor points and do not diminish the overall quality and significance of the study. I therefore recommend publication after minor revisions.

Reviewer #3: General Comments:

This manuscript investigates how musical encounters in healthcare settings enhance experiences of connection across individual, professional, and organizational levels. The ethnographic approach and the utilization of the "emotional process form" offer nuanced qualitative insights. However, the manuscript contains critical citation errors, issues with journal policy compliance, and ethical ambiguities that must be addressed before any further consideration.

Major Revisions:

1. Critical Citation Mismatch:

In lines 150-151, the authors state that musical encounters foster deep and multifaceted experiences of connection, citing "Simpson et al. [28]" as a reference. However, Reference 28 in the reference list is a medical meta-analysis titled "A meta-analysis of the association between adherence to drug therapy and mortality." This is an entirely unrelated medical paper that has no relevance to music or arts in health. The authors must rigorously audit their entire manuscript and correct this severe error.

2. Data Availability Policy Non-Compliance:

PLOS ONE maintains a strict data availability policy. The authors have stated that the data are not publicly available to protect participant privacy and have only provided metadata on DataverseNL. Under PLOS ONE guidelines, if raw data cannot be shared publicly, the authors MUST provide a non-author, independent institutional contact (e.g., a research ethics committee or data access committee) to which future researchers can apply for data access. Relying solely on metadata or a personal repository link is insufficient.

3. Ethics and Consent Clarification:

The authors state that the study required no ethical review (IRB) because it is non-medical. However, since the study involves human participants undergoing observations and interviews, a formal institutional waiver or exemption status should be verified. Please clarify whether a formal exemption was officially granted by the university's ethics board. Furthermore, please justify why verbal informed consent was preferred over written consent, and explain how the voluntariness of vulnerable populations (e.g., patients in general hospitals or residents in care homes) was protected.

Minor Revisions:

1. Typographical Error in References:

In Reference 30 (line 584), the publication year for the Netherlands Code of Conduct for Research Integrity is incorrectly printed as "1018" instead of "2018." Please correct this.

2. Methodological Limitation in Discussion:

The authors noted that they were restricted to interviewing only the professionals involved, thereby omitting the direct voices of patients, residents, and uninvolved staff. This limitation should be integrated more deeply into the Discussion section, reflecting on how the absence of these core perspectives might constrain the completeness of the "Connective experiences" matrix.

Reviewer #4: Live music encounter within healthcare system with the argument that it fosters connection is the main thrust of this research. This is supported with relevant literature, theoretical concepts and quantitative analysis. However without active participation of the patients through interview for data gathering, there is a gap regarding determination of the depth of this musical encounter and whether it fosters connection among the patients or with the care giver and musician. I believe this is an important aspect that should not be overlooked, it should be a tripartite connection, musician, healthcare professionals and patients but what is discussed here is within musician and healthcare professionals. If patients experience cannot be ascertained; this should be reflected at the onset through a review of the title, abstract and the research question(s). Therefore, we should be looking at- Live music encounters within healthcare systems and how it fosters connections between musicians and healthcare professionals and how healthcare workers react to music in respect of carrying out their daily tasks to their patients.

**********

what does this mean?). If published, this will include your full peer review and any attached files.

If you choose “no”, your identity will remain anonymous but your review may still be made public.

**Do you want your identity to be public for this peer review?**  For information about this choice, including consent withdrawal, please see our Privacy Policy

Reviewer #1: **Yes:**  Dr. Hu Yan

Reviewer #2: **Yes:**  ALBERTO BENTOGLIO

Reviewer #3: No

Reviewer #4: No

**********

[NOTE: If reviewer comments were submitted as an attachment file, they will be attached to this email and accessible via the submission site. Please log into your account, locate the manuscript record, and check for the action link "View Attachments". If this link does not appear, there are no attachment files.]

To ensure your figures meet our technical requirements, please review our figure guidelines: https://journals.plos.org/plosone/s/figures

You may also use PLOS’s free figure tool, NAAS, to help you prepare publication quality figures: https://journals.plos.org/plosone/s/figures#loc-tools-for-figure-preparation.

NAAS will assess whether your figures meet our technical requirements by comparing each figure against our figure specifications.

# Author comment: Author response to Decision Letter 1

**DOI:** 10.1371/journal.pone.0359306.r002

13 Aug 2026

Response to Academic Editor and Reviewers

Manuscript title: The Precarious Nature of Musical Encounters in Healthcare: An Ethnographic Multi-Case study Focusing on the Perspectives of Care Professionals and Musicians (previous title: The Precarious Nature of Musical Encounters in Healthcare: An Ethnographic Multi-Case Study)

Journal: PLOS ONE

Manuscript ID: [PONE-D-26-13817] - [EMID:de7335860df2f2c9]

Authors: Monique Bussmann, Mieke Cardol, Marieke Groot, Chris Kuiper

Dear Editor, Assoc. Prof. Phakkharawat Sittiprapaporn, Ph.D,

Thank you for handling our manuscript The Precarious Nature of Musical Encounters in Healthcare: An Ethnographic Multi-Case Study Focusing on the Perspectives of Care Professionals and Musicians, PONE-D-26-13817 (previous title: The Precarious Nature of Musical Encounters in Healthcare: An Ethnographic Multi-Case Study), and for the Reviewers’ careful reading.

We have revised the manuscript to address all comments. The main changes involve:

• Clarifying the ethical and legal restrictions on sharing a de-identified data set, as well as the authorities responsible for imposing these restrictions;

• Clarifying the empirical dataset, consolidating themes and strengthening conceptual coherence within the findings;

• Moderating the claims regarding the scope of the findings and the study’s contribution.

A point-by-point response follows; all page and line numbers refer to the revised manuscript with tracked changes. For clarity, we handle the comments of every reviewer in order of the review and reproduce each comment followed by our response and a brief indication of where changes are made in the manuscript.

We have labeled the Comments as follows:

• Editorial Comment

• Reviewer #1: Comments 1.1-1.19

• Reviewer #2: Comments 2.1-2.3

• Reviewer #3: Comments 3.1-3.4

• Reviewer #4: Comment 4.1

Reviewers’ comments are shown in italic, our responses in plain text.

Given the Reviewers’ request for careful technical editing, which led to numerous minor revisions that may obscure the more substantive changes to the manuscript, we have prepared two additional revision documents for your convenience:

• The revised manuscript, showing the revised text fragments while excluding tracked changes and minor technical edits (Revised Manuscript New Texts Marked in Red).

• A schematic overview of all comments and our responses (Scheme Response).

Finally, we have added an attachment to this letter as part of our response to the Editorial Comment (Statement_REC-RUAS_M.Bussmann).

________________________________________

1. Response to Academic Editor – Data availability and ethics prompt

Editorial Comment:

If there are ethical or legal restrictions on sharing a de-identified data set, please explain them in detail (e.g., data contain potentially identifying or sensitive patient information, data are owned by a third-party organization, etc.) and who has imposed them (e.g., a Research Ethics Committee or Institutional Review Board, etc.). Please also provide contact information for a data access committee, ethics committee, or other institutional body to which data requests may be sent.

Response:

This Editorial Comment – and similar comments from reviewers – prompted us to reconsider how we described the ethical issues in the manuscript and to clarify these issues in the new manuscript, accentuating the ethnographical design of the study, the specific research conditions at that time, and the focus on the perspectives of professionals (musicians and care professionals), as reflected in the new, expanded title of the manuscript (see also Reviewer #4’ Comment 4.1). We discussed the revisions with the university’s data officers, who verified their advice with the administrators of the DataverseNL repository platform. Furthermore, we consulted the chair of the university’s Research Ethics Committee for advice, who provided a statement supporting the ethical procedures we followed (see Attachment ‘Statement_REC-RUAS_M.Bussmann’).

Revisions:

p12: ‘All data, including interviews, observations, reports, andforms, as well as all analysis steps were securely stored in SURF Research Drive. Metadata were published on the repository platform DataverseNL (https://dataverse.nl/dataset.xhtml?persistentId=doi:10.34894/FBHF2Z). In accordance with the platform’s decision tree (https://zenodo.org/records/18390982), the data were assessed and deemed ineligible for publication, therefore only the metadata were published.’

p14: ‘Although RUAS aims for research data to be publicly available wherever possible, according to the prevailing data protection and confidentiality requirements, researchers of the university are not permitted to publicly share full transcripts or field notes, because of its contextual richness which makes full de-identification impossible without severely compromising the scientific value of the dataset and the participants’ consent.’

p14: ‘The musical encounters were initiated as innovative activities by the participating care institutions, rather than as a study intervention. Consequently, the researcher did not intervene in the natural setting, which is consistent with the ethnographic research design. As no data were collected directly from patients or residents, and no personal or health-related data were gathered at all, the study did not require medical-ethical approval. At the time the research design was reviewed (2019), RUAS did not yet have a Research Ethics Committee. The secretary of the current committee can be approached with requests for data access (e-mail eco@hr.nl).’

________________________________________

2. Response to Reviewer #1

We thank Reviewer #1 for the detailed and thoughtful comments and for recognizing the potential contribution of our study. We respond to each point below.

Formatting, Language, and Reference Issues

Comment (1.1): The manuscript requires careful technical editing before it can be considered for publication. There are numerous spelling, grammatical, formatting, and reference-list problems throughout the paper. While some of these issues may appear minor individually, their accumulation weakens the overall professionalism and credibility of the manuscript.

Response: Prompted by the Reviewers’ comments we have improved the writing by addressing the Reviewers’ comments, by thoroughly rereading the text and making the necessary corrections, and finally having it edited by a native English speaker.’

Comment (1.2): Several clear spelling and typographical errors should be corrected. For example, “review bord” should be “review board”; “Healtcare Settings” should be “Healthcare Settings”; “KNAW, 1018” appears to be a mistaken year and should presumably be “KNAW, 2018”; and the acknowledgement sentence “We like to thank...” should be revised to “We would like to thank...” or another standard formulation. There are also grammatical errors such as “Initial unease appear to play...” and “The findings also connects...”, both of which require correction.

Response: We have addressed these specific issues and carefully checked the manuscript for other errors correcting those identified.

Comment (1.3): The manuscript also contains formatting inconsistencies, including irregular spacing around dashes, inconsistent punctuation, and a visible double-parenthesis error in “(Table 3)”. These issues should be addressed systematically rather than corrected only locally. Since the paper relies heavily on tables and thematic matrices, table formatting should also be checked carefully to ensure that all headings, categories, and subthemes are clear and consistently presented.

Response: We have systematically addressed formatting inconsistencies in both the text and the tables.

Comment (1.4): The reference list also needs thorough checking. Some entries appear incomplete, inconsistent, or potentially inaccurate. For example, one DOI appears to be repeated in a way that may not correspond to the cited article, and at least one reference seems only weakly connected to the claim it is used to support. The authors should verify every reference against the source text, ensure that author names, years, titles, journal details, page ranges, and DOIs are correct, and bring the reference list into full compliance with the journal’s style.

Response:

• We have thoroughly checked both the reference list and all in-text references, making corrections, such as the DOI’s in question.

• We have checked the correspondence between the text and the cited sources and found that some links were indeed tenuous, although they could be justified because they are cited in the Scoping Review on Arts and Health [13]. To strengthen the manuscript and avoid overinterpretation, we decided to remove these references (Ayca et al., Crawford et al., Kaser et al., and Simpson et al.).

• We have incorporated some additional references, including Viola et al. [4], van de Berg et al. [6], Thompson [19], Fancourt et al. [28], Rosenberg [30], Tong et al. [31], and De Witte et al. [37]. Some of these were included to update and strengthen the manuscript, whereas others were added in response to Reviewers’ comments (discussed later in the response). Finally, we revised the reference list to conform to the Vancouver style required by PLOS One.

________________________________________

Methodological Transparency and Qualitative Reporting Standards

Comment (1.5): However, the methods section does not yet provide enough detail about the empirical dataset. The manuscript states that the researcher used open observations, open interviews, reflection reports, and an elicitation technique based on an emotional process form. It also states that data were compiled into a single database and analyzed in Atlas.ti. However, the reader is not given sufficiently clear information about the number of interviews, number and type of participants, number of observations, duration of fieldwork, number of reflection reports, number of emotional process forms, or the distribution of these materials across the three cases.

Response:

• To provide the requested details about the empirical dataset, we have made two revisions. First, we expanded Table 1 ‘Overview cases’ (p9-10) by adding a column that specifies the duration of each case. Second, we added Table 2 ‘Database: Cases and Data’ (p11-12) which provides detailed information on the participants and data for each case.

• To clarify the data analysis, we added the following information: ‘Within each case, data collection continued until no substantive new themes emerged in the observations and interviews, or until institutional access restrictions due to the COVID-19-pandemic made further fieldwork impossible.’ (p11)

And:

‘The data were compiled into a single database and qualitatively analyzed using Atlas.ti. The analysis followed an inductive process of constant comparison: open coding identified meaningful occurrences and participant interpretations, followed by axial coding, using the group code function in Atlas.ti.’ (p12)

Comment (1.6): ‘This lack of detail makes it difficult to assess the depth and balance of the evidence. I recommend that the authors add a clear data overview table showing, for each case, the types of data collected, number of participants, participant roles, number of interviews, number of observations, and any supplementary materials used. This would significantly improve the transparency and credibility of the qualitative design. Since this is a qualitative ethnographic multiple-case study, the authors should also ensure that the manuscript fully follows appropriate qualitative reporting standards, including the COREQ checklist mentioned in the manuscript, particularly regarding participant characteristics, recruitment, data volume, researcher reflexivity, analytic procedures, and the adequacy of the empirical material.’

Response: We refer to our response above (Comment 1.5).

We have reapplied the COREQ checklist [31] to ensure that the manuscript fully complies with qualitative reporting standards. Consequently, we have added:

• Table 2 ‘Database: Cases and Data’ (p9-10), describing participant characteristics and data volume;

• More details on participant recruitment (p8). ‘The individual participants (such as project leaders, care professionals and musicians) were recruited by the respective facilitators, provided with information about the study, and invited to participate voluntarily.’;

• ’More detailed information on the data collection and analytic procedures (p11): ‘Within each case, data collection continued until no substantive new themes emerged in several of the observations and interviews, or until institutional access restrictions due to the COVID-19-pandemic made further fieldwork impossible.’;

• A section Fieldwork (p13) describing the researcher’s reflexivity and the adequacy of the empirical material:

‘The study coincided with the COVID-19 pandemic, which demanded the full attention of healthcare institutions and made them highly reluctant to grant access to musicians and, even more, to researchers. Consequently, the researcher’s access for data collection was largely limited to musicians, project leaders/facilitators and directly involved staff members, rather than to patients/residents, care staff and managers.

As a result, the dataset does not include the direct voices of patients/residents and healthcare professionals who were not directly involved, or board members. However, the musicians, project leaders/facilitators, and professionals who participated directly proved both willing and able to represent the perspectives of patients/residents, other professionals, and managers. It can be concluded that this has limited ability to answer the research question with the desired comprehensiveness. Nevertheless, these specific circumstances provided particularly rich insights into the dynamics at the professional level, as well as at the other levels.’

Comment (1.7): The authors should also clarify the role of the researcher in the field. The manuscript mentions that the researcher sometimes participated modestly, for example by singing or assisting participants, and at other times observed unobtrusively. This is appropriate for ethnographic work, but it requires more reflexive discussion. The authors should explain how the researcher’s professional background, existing networks, and occasional participation may have shaped access, observation, interpretation, and participant responses.

Response: We refer to point 4 in our response to Comment (1.6) above, particularly the following text in the Fieldwork section regarding the researcher’s reflexivity (p13-15): ‘Being a former nurse and project manager in healthcare, as well as an amateur musician herself, the researcher is acquainted with the worlds of experience of respondents and capable of getting access, in-depth observation and interviewing, analyzing, and interpreting. As an experienced qualitative researcher, she continuously reflected on which attitude would best preserve the naturalness of the situation, and remained aware that, despite these efforts, her presence may have influenced the participants’ behavior.’ (p13)

________________________________________

Ethics and Data Reporting

Comment (1.8): The ethics statement requires urgent clarification. The submission information states “N/A” for ethics, while the manuscript clearly involves human participants and observations in healthcare environments. Because PLOS ONE requires appropriate approval or a clear justification for studies involving human participants, including observational studies and studies using personal data, the ethics statement should not simply be marked as “N/A” without a more precise explanation. The manuscript explains that the study was not medical in nature and that the institutions involved, together with the university of applied sciences, determined that no medical IRB review was required. However, this explanation is not sufficient in its current form.’

Comment (1.9): The authors should clarify whether a formal ethics review exemption or waiver was obtained, which institution or body made this determination, and whether there is a documented decision. They should also explain more precisely how informed consent was obtained, especially for observations in healthcare settings and for any participants who were not directly interviewed. The manuscript states that verbal informed consent was captured in interview recordings, but it remains less clear how consent was handled for observed musical encounters involving patients, residents, visitors, or staff who may not have been interviewed.

This issue is especially important because the study takes place in healthcare and residential care environments, where questions of vulnerability, privacy, and consent are particularly sensitive. Even if the study is not a medical intervention, it still involves human participants and should therefore provide a more robust ethics statement.

Comment (1.10) The data availability statement also needs clarification. I understand that full qualitative data may not be publicly shareable because of participant privacy and the sensitivity of healthcare and residential care settings. However, the current statement should specify whether any controlled access to the underlying data is possible, which institution or committee is responsible for access requests, what criteria would be used to assess such requests, and who qualified researchers should contact. If no access to the underlying data is possible, this should be clearly justified in relation to participant consent, privacy protection, and institutional requirements. This clarification is important for ensuring compliance with PLOS ONE’s data availability expectations while also protecting participants’ confidentiality.

Response: In response to these three Comments, we refer to our response to the Editorial Comment above.

________________________________________

Analytical Structure and Presentation of Findings

Comment (1.11) However, the current results section is overly fragmented. The analysis includes 34 subthemes across two major themes and three levels. While this shows empirical richness, it also produces a catalogue-like structure. Some subthemes are described only briefly, and the relationship between them is not always sufficiently analyzed. The paper would benefit from consolidating overlapping subthemes and giving more interpretive attention to the most important mechanisms.

Response: We have consolidated overlapping subthemes, as presented in the revised Table 3 ‘Experiences within musical encounters’(p16), revised Table 4 ‘Experiences among involved professionals’ (p20-21), and revised Table 5 ‘Experiences within the broader organization’ (p25).

Furthermore, we have given more attention to the most important mechanisms by adding a subsection entitled ‘Interpretations and Implications’ to each results subsection (resp. p19-20, p23-24, and p28-29).

Comment (1.12): In particular, the authors should make clearer how threshold experiences relate to connective experiences. Are threshold experiences necessary preconditions for connection? Are they barriers that must be overcome? Do some thresholds generate connection while others prevent it? Are the dynamics different at the level of the musical encounter, professional collaboration, and organizational embedding? At present, these relationships are sometimes asserted rather than fully demonstrated.

Response: We have addressed this comment as follows in the three subsections ‘Interpretations and Implications’ mentioned above (Response to Comment 1.11) and in the section Conclusions:

• Experiences within the musical encounters (p20): ‘Although uncomfortable feelings often precede connective sensations, they are not a necessary precondition. Connections frequently occur without a preceding threshold experience. At the same time, initial uncomfortable feelings can act as a barrier to connection; conversely, they may also enhance and deepen them. Within musical encounters, most listeners appear willing and motivated to overcome their initial uneasiness and to engage in connective experiences.’;

• Experiences among involved professionals (p24): ‘At this level as well, threshold experiences do not seem to be a necessary condition for experiences of connection. When they occur, they may either obstruct or enrich them. The findings at this level, which focus on the professionals’ perspective on their own experiences, constitute the central contribution of this study.’;

• Experiences within the broader organization (p29): ‘In contrast to the two levels discussed above, the threshold experiences at this level, characterized as management and board members ‘staying out’, may function both as barriers to and preconditions for experiences of organizational connection. The empirical evidence and implications of this interpretation are relatively limited, as the findings are primarily based on the experiences and interpretations of project leaders, care professionals and musicians, supplemented with observations of the researcher. Key actors at the organizational level were only minimally engaged in both the music project and the study. Nevertheless, these findings may provide valuable directions for both implementation and future research.’;

• Conclusions (p34): ‘Some thresholds are so low that they may remain unnoticed, while others are clearly evident. At times, threshold experiences are productive, fostering connective experiences (e.g. within musical encounters), sometimes even serving as a precondition for connection to occur. At other times, they prevent experiences of connection altogether. Consistent with the arts therapy literature, these dynamics are inherently personal, relational, and situational, and the how and why still remains unclear [37].’

Referring to Reviewer #1’s own academic thoughts (1.13):We are very grateful to Reviewer #1 for providing additional suggestions to strengthen the conceptual coherence and readability of the manuscript, drawing on their own academic expertise. We gave these suggestions careful consideration, which is reflected in our responses to Comments (1.11) and (1.12).

________________________________________

Theoretical Framing and Positioning within Arts-in-Health, Music Therapy, and Creative Arts Therapy Research

Comment (1.14): However, in its current form, the theoretical framework is overextended. The manuscript moves quickly between different bodies of literature, and not all of them are equally integrated into the empirical analysis. As a result, the reader may find it difficult to determine which theoretical concepts are central to the manuscript’s original contribution and which are mainly contextual or supportive.

Response: We have added the following text to the Discussion (p31): ‘The theoretical view on human beings as inherently musical and caring [16, 18] and the ethical and aesthetic perspective on healthcare [18,19,20] provide lenses through which the findings can be interpreted and reflected upon. Spitzer’s anthropological perspective on music making [16], and Tronto’s ethical-political perspective on the distribution of care responsibilities [18] are central to deepening the understanding of the observed dynamics. As participants’ aesthetic experiences were not explored in depth, the aesthetic perspectives of Thompson [19], De Kock [20] and Schiuma [21] on healthcare and its institutions, play a more supportive role, enriching and deepening the findings.’

Furthermore, we have reduced the emphasis on the work of moral philosopher Walker in subsection ‘Ethical an aesthetic perspectives on healthcare’ (p6) and on feminist philosopher Butler in the subsection ‘Overarching construct: precariousness’ (p29).

Comment (1.15): p9 The authors may also consider using music therapy and arts therapy literature to refine the meaning of “threshold experiences.” In the current manuscript, threshold experiences include discomfort, embarrassment, uncertainty, unfamiliarity, role ambiguity, professional difference, work pressure, and organizational resistance. These are important findings, but they could be theorized more precisely. In music therapy and arts therapy, discomfort or vulnerability is not always a negative barrier; it can sometimes be part of the process through which emotional expression, trust, and connection become possible.

Comment (1.16): p9 However, this is not always the case. Some thresholds may be artistically or relationally productive, while others may be institutionally obstructive. This distinction is central to the manuscript’s own argument about precariousness. At the level of the musical encounter, uncertainty and sensitivity may be part of the delicate artistic process. At the organizational level, however, lack of funding, unclear responsibility, weak managerial commitment, and short-term policymaking are less likely to be productive thresholds; rather, they place the practice at risk. The authors should make this distinction more explicit.

Response to both comments: Here, we refer to our responses on Comments (1.11) and (1.12). Furthermore, we have added a reference to De Witte et al. [37] regarding the mechanisms of change in the creative arts therapies (p34).

________________________________________

Scope of Claims and Contribution

Comment (1.17) The manuscript makes several important claims about the potential of musical encounters to support connection, professional enrichment, interprofessional collaboration, employee well-being, and organizational innovation. These are valuable directions. However, some claims should be moderated because the empirical material does not always fully support the broader implications. For example, the discussion suggests that musical encounters may contribute to staff retention, employee-employer relationships, and healthcare system sustainability. These are plausible implications, but they are not directly demonstrated by the data in the current version.

Response: We agree with the reviewer and have added the following text to the Discussion (p31): ‘The findings also may connect musical encounters to broader healthcare challenges. For instance, shared musical enjoyment and inspiration may support quality of care, job satisfaction and staff retention, while improved employee-employer relationships could inform more efficient and effective care structures. These remain hypothetical implications rather than outcomes demonstrated in the present study. However, recognizing the potential contribution of musical encounters to future institutional challenges may enable managers and board members to lead innovations more effectively.’

Furthermore, we added nuance to previous assertions (p32): ‘Finally, the study may reinforce aesthetic perspectives on healthcare. Echoing Thompson [19] and De Kock et al. [20] the findings may indicate that musicians can enhance the quality of both care and care work, through evoking aesthetic sensations. These findings also may resonate with Schiuma’s [21] ideas about the value of aesthetic sensations in organization development, suggesting that similar principles could help care and cultural institutions navigate transitions. Despite participants acknowledging this potential, assumptions that musical encounters would benefit the organization or the wider healthcare system remained largely cognitive and aspirational in this study, rather than empirically realized.’

Comment (1:18): p10 The authors should distinguish more clearly between findings, interpretations, and implications. The strongest findings concern experiences of connection and threshold within specific musical encounters and professional contexts. Broader organizational or policy implications should be framed more cautiously, especially given the limited number of cases and the fact that patients, residents, uninvolved staff, and senior managers are not directly or systematically represented as participant groups.

Response: Here, we refer here to our responses to Comments (1.12) and (1.17).

________________________________________

Others – Use of AI Tools and Language Editing

Comment (1.19): p10 The disclosure that Microsoft 365 Copilot was used for English grammar checking and word-count reduction is appropriate in principle. However, the manuscript still contains numerous grammatical, typographical, formatting, and reference errors. The manuscript still requires careful human proofreading and consistency checking.

Disclosure of Microsoft 365 Copilot use is appropriate, but manuscript still needs careful human proofreading.

Response: Here, we refer hto our responses on Comments (1.1 to 1.4) and (2.1 to 2.2).

________________________________________

General Appreciation

Reviewer comment:

The manuscript could make a meaningful contribution to research on arts in health, relational care, healthcare work environments, and transdisciplinary collaboration.

Response:

We thank Reviewer #1 for these encouraging remarks. We hope that the revisions in language, methods, ethics, analysis, and theory strengthen the manuscript’s contribution in the ways suggested.

________________________________________

3. Response to Reviewer #2

We are grateful to Reviewer #2 for evaluating the manuscript as extremely valuable, well written, and suitable for publication, and for providing helpful suggestions for further improvement.

Comment: (2.1): In the Discussion, some conceptual passages concerning “connection” and “precariousness” could be slightly streamlined to avoid occasional repetition.

Response: We thoroughly revised the Discussion (p30-34), in response to several comments. In the revised version we avoided repetition of key concepts.

Comment (2.2): p11 A final careful language editing by a native English speaker or professional editor may help improve fluency in a few passages, although the manuscript is already fully intelligible and generally written in good academic English.

Response: Here, we refer here to our response to Reviewer #1’s Comment (1.1) above.

Comment (2.3): p11 Although the methodological limitations are acknowledged appropriately, the manuscript could further clarify the implications of the restricted access to certain participant groups (patients, uninvolved staff, administrators) for the transferability of the findings.

Response: Here, we refer here to our response to Reviewer #4’s Comment (4.1) below.

________________________________________

4. Response to Reviewer #3

We thank Reviewer #3 for the careful reading and for highlighting important issues regarding missing direct voices, citation errors and ethical ambiguities. We have reviewed it carefully and hope that we have been able to address any concerns.

________________________________________

Major Revisions

Comment (3.1) titled ‘Critical Citation Mismatch’: In lines 150-151, the authors state that musical encounters foster deep and multifaceted experiences of connection, citing "Simpson et al. [28]" as a reference. However, Reference 28 in the reference list is a medical meta-analysis titled "A meta-analysis of the association between adherence to drug therapy and mortality." This is an entirely unrelated medical paper that has no relevance to music or arts in health. The authors must rigorously audit their entire manuscript and correct this severe error.

Response: Here, we refer to our responses to Comments (1.1 to 1.4), particularly Comment (1.4), which discusses the reference to Simpson et al.

Comment (3.2) titled ‘Data Availability Policy Non-Compliance’. PLOS ONE maintains a strict data availability policy. The authors have stated that the data are not publicly available to protect participant privacy and have only provided metadata on DataverseNL. Under PLOS ONE guidelines, if raw data cannot be shared publicly, the authors MUST provide a non-author, independent institutional contact (e.g., a research ethics committee or data access committee) to which future researchers can apply for data access. Relying solely on metadata or a personal repository link is insufficient.

Response: Here, we refer to our response to the Editorial Comment above.

Comment (3.3) titled ‘Ethics and Consent Clarification’. The authors state that the study required no ethical review (IRB) because it is non-medical. However, since the study involves human participants undergoing observations and interviews, a formal institutional waiver or exemption status should be verified. Please clarify whether a formal exemption was officially granted by the university's ethics board. Furthermore, please justify why verbal informed consent was preferred over written consent, and explain how the voluntariness of vulnerable populations (e.g., patients in general hospitals or residents in care homes) was protected.

Response: In addition to our response to the Editorial Comment we have provided following extra information in the revised manuscript.

• Referring to the section ‘Ethical Considerations’ (p13-15), we have added: The musical encounters were initiated as innovative activities by the participating institutions, rather than as a study intervention. Consequently, the researcher did not intervene in the natural setting, which is consistent with the ethnographic research design.’ As well as the sentence: ‘All verbal informed consents were captured in the recordings of the interviews, that in most cases were conducted online due to COVID-19-measures.’;

• To clarify the impact of COVID-19 restrictions on the research process (e.g., the inability to conduct face-to-face interviews and obtain written consent), we have referred to these restrictions more explicit throughout our revision process.;

• Referring to the Data Collection, we have added the following information (p11): In cases where COVID-19 restrictions prevented observation, participating musicians, welfare and care professionals submitted reflection reports.’ As well as the sentence: ‘Within each case, data collection continued until no substantively new themes emerged in the observations and interviews, or until institutional access restrictions due to the COVID-19-pandemic made further fieldwork impossible.’;

• Referring to Ethical Considerations (p13-15), we have added: ‘…that in most cases were conducted online due to COVID-19-measures.’ to the sentence ‘All verbal informed consents were captured in the recordings of the interviews’;

• Finally, referring to the methodological discussion (p33-34) we added following information: In all three cases, some musical performances were cancelled or could not be observed due to COVID-19 measures. In addition, these restrictions prevented the researcher from interviewing patients/residents and care professionals who were not directly involved in the musical encounters.’

________________________________________

Minor Revisions

Comment (3.4): The authors noted that they were restricted to interviewing only the professionals involved, thereby omitting the direct voices of patients, residents, and uninvolved staff. This limitation should be integrated more deeply into the Discussion, reflecting on how the absence of these core perspectives might constrain the completeness of the "Connective experiences" matrix.

Response: Addressing this revision point:

• In the new section ‘Fieldwork’ (p13) we added the following phrase: ‘The study coincided with the COVID-19 pandemic, which demanded the full attention of healthcare institutions and made them highly reluctant to grant access to musicians and, even more, to researchers. Consequently, the researcher’s access for data collection was largely limited to musicians, project leaders/facilitators and directly involved staff members, rather than to patients/residents, care staff and managers. As a result, the dataset does not include the direct voices of patients/residents and healthcare professionals who were not directly involved, or board members. However, the musicians, project leaders/facilitators, and professionals who participated directly proved both willing and able to represent the perspectives of patients/residents, other professionals, and managers. It can be concluded that this has limited ability to answer the research question with the desired comprehensiveness. Nevertheless, these specific circumstances provided particularly rich insights into the dynamics at the professional level, as well as at the other levels.’;

• In the subsection Methodological discussion (p33) we added the following text: ‘First, in all three cases, some musical performances were cancelled or could not be observed due to COVID-19 measures. In addition, these restrictions prevented the researcher from interviewing patients/residents and care professionals who were not directly involved in the musical encounters. Secondly, care professionals, often working under time constraints, were generally less inclined to engage in the musical encounters, focusing instead on clinical responsibilities. Managers and board members were even more detached. As a result, their perspectives are underrepresented, limiting the completeness and depth of the findings.’

Comment (3.5): ‘Typographical Error in References’. In Reference 30 (line 584), the publication year for the Netherlands Code of Conduct for Research Integrity is incorrectly printed as "1018" instead of "2018." Please correct this.

Response: We have corrected this.

________________________________________

5. Response to Reviewer #4

We thank Reviewer #4 for highlighting the important issue of patient participation and the scope of the study.

Comment (4.1): Without active participation of the patients through interview for data gathering, there is a gap regarding determination of the depth of this musical encounter and whether it fosters connection among the patients or with the care giver and musician. (…) If patients experience cannot be ascertained; this should be reflected at the onset through a review of the title, abstract and the research question(s).

Because we largely agree with this comment, we have:

• Adjusted the title of the manuscript (p1): ‘The precarious nature of musical encounters in healthcare. An ethnographic multi-case study focusing on the perspectives of care professionals and musicians, including the abstract and research question.’;

• Reflected on this limitation in subsection ‘Interpretations and implications’ (p20): ‘An empirical limitation is the absence of patients’ and residents’ direct voices, since the findings are based on the musicians’ and care professionals’ experiences and observations regarding musical performances, supplemented by the researcher’s observations. Nevertheless, this limitation is somewhat offset by the wealth of existing studies that have already documented patients’ and residents’ perspectives on the role and effects of music in care settings [1,2,3,4]’.

• Discussed this limitation in the Methodological Discussion (p30-34):

‘This study faced several methodological limitations that shaped its scope and findings. First, in all three cases, some musical performances were cancelled or could not be observed due to COVID-19 measures. In addition, these restrictions prevented the researcher from interviewing patients/residents and care professionals who were not directly involved in the musical encounters. Secondly, care professionals, often working under time constraints, were generally less inclined to engage in the musical encounters, focusing instead on clinical responsibilities. Managers and board members were even more detached. As a result, their perspectives are underrepresented, limiting the completeness and depth of the findings.

It can be concluded that the exploratory nature of the study, the limited number of cases, and restricted access to events and participants due to the COVID-19 measures, the transferability and broader implications of the findings are limited. However, the methodology’s strength lies in its contextual richness, revealing the importance of the organizational context surrounding the traditional tripartite relationship between patient, professional, and musician in musical encounters in healthcare.’;

• incorporated it in the recommendations for further research in the Conclusions (p35): ‘Further research could explore new cases and methodological approaches to validate and extend these findings, particularly by incorporating contexts and perspectives that were underrepresented in the present study, including the direct voice of patients/residents, care professionals who are not directly involved in musical activities, and the perspectives of managers and board members.’

________________________________________

6. Closing Statement

We hope these revisions resolve the Editor’s and Reviewers’ concerns and look forward to your decision.

Sincerely,

Monique B.M. Bussmann, on behalf of all co-authors

Attachment Submitted filename: Response to Reviewers.docx

# Peer review: Decision Letter 1

**DOI:** 10.1371/journal.pone.0359306.r003

**Authors:** Assoc. Prof. Phakkharawat Sittiprapaporn

14 Sep 2026

The precarious nature of musical encounters in healthcare: An ethnographic multi-case study focusing on the perspectives of care professionals and musicians

PONE-D-26-13817R1

Dear Dr. Bussmann,

We’re pleased to inform you that your manuscript has been judged scientifically suitable for publication and will be formally accepted for publication once it meets all outstanding technical requirements.

Within one week, you’ll receive an e-mail detailing the required amendments. When these have been addressed, you’ll receive a formal acceptance letter and your manuscript will be scheduled for publication.

An invoice will be generated when your article is formally accepted. Please note, if your institution has a publishing partnership with PLOS and your article meets the relevant criteria, all or part of your publication costs will be covered. Please make sure your user information is up-to-date by logging into Editorial Manager at Editorial Manager® and clicking the ‘Update My Information' link at the top of the page. For questions related to billing, please contact billing support.

If your institution or institutions have a press office, please notify them about your upcoming paper to help maximize its impact. If they’ll be preparing press materials, please inform our press team as soon as possible -- no later than 48 hours after receiving the formal acceptance. Your manuscript will remain under strict press embargo until 2 pm Eastern Time on the date of publication. For more information, please contact onepress@plos.org.

Kind regards,

Assoc. Prof. Phakkharawat Sittiprapaporn, Ph.D.

Academic Editor

PLOS One

Reviewer's Responses to Questions

**Comments to the Author**

Reviewer #1: All comments have been addressed

Reviewer #2: (No Response)

Reviewer #4: All comments have been addressed

**********

2. Is the manuscript technically sound, and do the data support the conclusions?

Reviewer #1: Yes

Reviewer #2: Yes

Reviewer #4: Yes

**********

3. Has the statistical analysis been performed appropriately and rigorously?

Reviewer #1: N/A

Reviewer #2: N/A

Reviewer #4: N/A

**********

4. Have the authors made all data underlying the findings in their manuscript fully available?

The PLOS Data policy

Reviewer #1: No

Reviewer #2: No

Reviewer #4: No

**********

5. Is the manuscript presented in an intelligible fashion and written in standard English?

Reviewer #1: Yes

Reviewer #2: Yes

Reviewer #4: Yes

**********

Reviewer #1: Thank you for the careful and substantial revision of the manuscript. I have reviewed both the revised version and the authors' detailed responses to the previous round of comments. In my view, the major concerns raised during the earlier review have now been adequately addressed.

The revised manuscript is considerably clearer in its methodological reporting, empirical scope, analytical structure, and theoretical positioning. In particular, the clarification of the dataset, the consolidation of the thematic findings, the more explicit distinction between threshold and connective experiences, the improved discussion of ethical and data-access issues, and the moderation of claims beyond the direct evidence have strengthened the manuscript substantially. I also appreciate the clearer acknowledgement of the limitations resulting from the absence of direct patient/resident perspectives and the limited representation of senior organizational actors.

I therefore consider the manuscript suitable for publication.

Nevertheless, I would like to present some reflections on the future research of the authors, rather than requests for further revision of the current paper.

One very promising direction of the study is the suggestion proposed by Tronto's care ethics: Musicians in healthcare may be viewed not only as artists but potentially as "artistic caregivers". This idea has been confirmed to some extent. It is no longer about whether music has a good effect; rather, the question has shifted to when an artistic experience can itself be considered a kind of care.

In the future, we will study how an artist comes to have such a role. For example, one wants to know how much attention, response speed, situational awareness, reciprocity, professional respect and organisational support are required. It can help to organise the division of labour among performers, artists' caregivers, care staff and music therapists. It should not be assumed that a musician present in a healthcare setting is automatically a caregiver; rather, artistic caregiving may be realized under specific relational and organisational circumstances.

A new theoretical path has been proposed in the manuscript for "precariousness". It can be seen that the reasons for precariousness are changing. Uncertainty, vulnerability, unfamiliarity and other shyness in musical gatherings are sometimes positive and can also be a type of closeness. Other types of organisational instability that threaten the continuity of the partnership are unstable funds, unclear responsibilities, weak management commitment, lack of power and short-term policies, rather than creating relational opportunities.

Therefore, future work will distinguish more clearly between "productive or relational precariousness" and "institutional or destructive precariousness". Therefore, it can be proposed that the foundation of stable arts-in-health practice should not necessarily be the complete elimination of precariousness, but rather the reduction of institutional vulnerability while retaining the openness, sensitivity, spontaneity and uncertainty required for deep artistic experience.

This also leads to a problem of institutionalisation. If the healthcare organisations officially recognize artistic caregiving, it will be given better support and more organised professional training. At the same time, excessive standardisation or a focus on results and efficiency in management may restrict the scope of artistic freedom and deep relational experience. The above possible "institutionalisation paradox" will be further explored in an extended study that investigates how arts-in-health initiatives evolve from temporary projects into organisational-embedded practices.

At a higher level, with the continuous blurring of boundaries among artistic creation, art performance, caregiving and therapeutic intervention, these problems will be even more severe. Developments in digitally mediated and AI-assisted musical practice may eventually offer a particularly interesting environment for testing the theoretical ideas in this paper. If an adaptive technological system can generate highly personalised musical experiences and produce feelings of comfort or connection, future research may need to explore whether care is primarily based on experienced outcomes, or whether reciprocity, embodied vulnerability, moral responsibility and the presence of another human being are constitutive elements of caregiving itself.

Therefore, at this time, the idea of precariousness may also be relevant to the long-term development of musical projects. It may also offer a way to explore what constitutes authentic relatedness in artistic care: perhaps the musician and the participant can be mutually responsive, both vulnerable to emotion and fully engaged in a meeting with no foreseeable end.

These problems are beyond the scope of this paper and should not be regarded as additional reasons for revision. Rather, I believe they are indications that the study has started a fruitful line of research with extensive prospects for future exploration at the intersection of arts in health, care ethics, professional practice, organisational studies and emerging technologies.

In short, I believe that the revised paper is now an excellent and publishable contribution to this field of research. Specifically, it has moved away from focusing solely on the effects of music to explore how relational, professional and organisational contexts shape meaningful musical experiences.

Reviewer #2: The revised manuscript presents original research and offers a well-motivated and carefully developed ethnographic multi-case study on musical encounters in healthcare settings. In my view, the study is technically sound, and the qualitative analysis is performed to an appropriate standard and described in sufficient detail. The conclusions are presented in a balanced way and are adequately supported by the data and materials discussed in the manuscript. I particularly appreciate the manuscript’s attention to the performative, relational, professional, and organizational dimensions of musical encounters, moving beyond a narrow understanding of music as a therapeutic stimulus. The distinction between “threshold experiences” and “connective experiences” is effective, and the use of “precariousness” as an overarching interpretive concept is convincing and productive. The revised version also appears to address the main methodological and editorial issues raised during review, including greater transparency regarding the empirical dataset, clarification of the ethical and legal restrictions on data sharing, and improvement of language, formatting, and references. The article is presented in an intelligible fashion and is written in standard English. Although the full underlying qualitative data are not publicly available, the authors have now provided a clear explanation of the ethical and legal restrictions, published metadata, and indicated an institutional contact for data access requests. I find this explanation acceptable. I have no specific concerns regarding research ethics, publication ethics, research integrity, or potential dual publication. Overall, my assessment is positive, and I recommend publication.

Reviewer #4: (No Response)

**********

what does this mean?). If published, this will include your full peer review and any attached files.

If you choose “no”, your identity will remain anonymous but your review may still be made public.

**Do you want your identity to be public for this peer review?**  For information about this choice, including consent withdrawal, please see our Privacy Policy

Reviewer #1: **Yes:**  Hu, Yan (Wenan)

Reviewer #2: No

Reviewer #4: No

**********

# Editor report: Acceptance letter

**DOI:** 10.1371/journal.pone.0359306.r004

**Authors:** Assoc. Prof. Phakkharawat Sittiprapaporn

PONE-D-26-13817R1

PLOS One

Dear Dr. Bussmann,

I'm pleased to inform you that your manuscript has been deemed suitable for publication in PLOS One. Congratulations! Your manuscript is now being handed over to our production team.

At this stage, our production department will prepare your paper for publication. This includes ensuring the following:

* All references, tables, and figures are properly cited

* All relevant supporting information is included in the manuscript submission,

* There are no issues that prevent the paper from being properly typeset

You will receive further instructions from the production team, including instructions on how to review your proof when it is ready. Please keep in mind that we are working through a large volume of accepted articles, so please give us a few days to review your paper and let you know the next and final steps.

Lastly, if your institution or institutions have a press office, please let them know about your upcoming paper now to help maximize its impact. If they'll be preparing press materials, please inform our press team within the next 48 hours. Your manuscript will remain under strict press embargo until 2 pm Eastern Time on the date of publication. For more information, please contact onepress@plos.org.

You will receive an invoice from PLOS for your publication fee after your manuscript has reached the completed accept phase. If you receive an email requesting payment before acceptance or for any other service, this may be a phishing scheme. Learn how to identify phishing emails and protect your accounts at https://explore.plos.org/phishing.

If we can help with anything else, please email us at customercare@plos.org.

Thank you for submitting your work to PLOS One and supporting open access.

Kind regards,

PLOS One Editorial Office Staff

on behalf of

Assoc. Prof. Dr. Assoc. Prof. Phakkharawat Sittiprapaporn

Academic Editor

PLOS One
